# *TaGW2*, a Good Reflection of Wheat Polyploidization and Evolution

**DOI:** 10.3389/fpls.2017.00318

**Published:** 2017-03-07

**Authors:** Lin Qin, Junjie Zhao, Tian Li, Jian Hou, Xueyong Zhang, Chenyang Hao

**Affiliations:** ^1^Crop Genomics and Bioinformatics Center and National Key Lab of Crop Genetics and Germplasm Enhancement, Nanjing Agricultural UniversityNanjing, China; ^2^Key Laboratory of Crop Gene Resources and Germplasm Enhancement, Ministry of Agriculture/The National Key Facility for Crop Gene Resources and Genetic Improvement/Institute of Crop Science, Chinese Academy of Agricultural SciencesBeijing, China

**Keywords:** *TaGW2*, genetic differentiation, grain size, nucleotide polymorphism, *Triticum aestivum*

## Abstract

Hexaploid wheat consists of three subgenomes, namely, A, B, and D. These well-characterized ancestral genomes also exist at the diploid and tetraploid levels, thereby rendering wheat as a good model species for studying polyploidization. Here, we performed intra- and inter-species comparative analyses of wheat and its relatives to dissect polymorphism and differentiation of the *TaGW2* genes. Our results showed that genetic diversity of *TaGW2* decreased with progression from the diploids to tetraploids and hexaploids. The strongest selection occurred in the promoter regions of *TaGW2-6A* and *TaGW2-6B*. Phylogenetic trees clearly indicated that *Triticum urartu* and *Ae. speltoides* were the donors of the A and B genomes in tetraploid and hexaploid wheats. Haplotypes detected among hexaploid genotypes traced back to the tetraploid level. *Fst* and π values revealed that the strongest selection on *TaGW2* occurred at the tetraploid level rather than in hexaploid wheat. This infers that grain size enlargement, especially increased kernel width, mainly occurred in tetraploid genotypes. In addition, relative expression levels of *TaGW2s* significantly declined from the diploid level to tetraploids and hexaploids, further indicating that these genes negatively regulate kernel size. Our results also revealed that the polyploidization events possibly caused much stronger differentiation than domestication and breeding.

## Introduction

Polyploidization has played an important role in the evolution of plant eukaryotes. Polyploids arise by chromosome doubling of an individual genome (autoploidy) or by chromosome doubling of hybrids between species whose chromosomes normally do not pair (allopolyploidy). Common or bread wheat (*Triticum aestivum* L., 2n = 6x = 42), which represents one of the best-characterized examples of polyploidization, evolved through two hybridization events (Marcussen et al., 2014). Common wheat consists of three sets (or genomes) of homologous chromosomes, named A, B, and D, with each composed of 7 chromosomes. Bread wheat evolved by two spontaneous hybridization events (Feuillet et al., 2008). Natural hybridization between diploid species *T. urartu* (2n = 2x = 14, AA) and an unknown B genome species, giving rise to a tetraploid species (*T. dicoccoides* L., 2n = 28, AABB), occurred about 500,000 years ago (Dvorák et al., 1993; Mori et al., 1995; Huang et al., 2002; Dvorak and Akhunov, 2005). The origin of the B genome has been a discussion topic for many years, and differences in viewpoint have hindered its elucidation (Sarkar and Stebbins, 1956; Kimber and Athwal, 1972; Dvorák and Zhang, 1990; Wang et al., 1997; Maestra and Naranjo, 1998; Huang et al., 2002). More recent studies generally support the view that *Aegilops speltoides* (2n = 2x = 14, SS) is the donor, or major contributor of the B genome (Petersen et al., 2006; Kilian et al., 2007). The second step hybridization took place 7,000–10,000 years ago between a tetraploid species and diploid *Ae. tauschii* (2n = 2x = 14, DD) (Kihara, 1944; McFadden and Sears, 1944), resulting in the bread wheat (2n = 6x = 42, AABBDD) (Kihara, 1944; Huang et al., 2002). Compared to other allopolyploids wheat is considered to be a relatively young polyploid. For this reason and its importance as a major food crop wheat has long been employed as a classical model for studying the process of allopolyploidization in crop plants.

Grain weight is an important domestication and breeding trait. Rice is an important crop model diploid plant and its yield genetics have been studied extensively (Xing and Zhang, 2010; Bai et al., 2012; Zuo and Li, 2014). The cloned *GW2* on rice chromosome 2S encodes a ubiquitin E3 ligase, whose deletion leads to increased grain width and weight, thereby improving yield (Song et al., 2007), but it was not strongly selected during domestication or in breeding (Lu et al., 2013). Research on wheat *GW2* homologs has been extensive, and includes gene cloning, functional marker development and elucidation of the genetic effects of each homolog (Su et al., 2011; Qin et al., 2014; Jaiswal et al., 2015). Expression analysis suggested that the *TaGW2* genes were constitutively expressed in different tissues (Su et al., 2011). Yang et al. (2012) identified a single-base insertion in the eighth exon of *TaGW2-6A* in the landrace Lankaodali. This caused premature termination and led to increased grain width and weight. However, RNAi results showed that the patterns of *TaGW2* regulation on grain development might be more complex (Bednarek et al., 2012; Hong et al., 2014). Simmonds et al. (2016) screened an EMS TILLING population of a tetraploid wheat cultivar “Kronos” and found that a *GW2-A1* mutant allele significantly increased thousand grain weight, grain width and grain length in both durum and bread wheats. These studies mainly focused on gene cloning, marker development, and expression analysis, whereas the evolution of *TaGW2s* during wheat polyploidization was not examined yet.

Nucleotide polymorphism and genetic differentiation of three *TaGW2* homologs in wheat and its ancestors and relatives were investigated in the present study, the aims of which were to: (1) determine the *TaGW2* nucleotide diversity at the genomic level in 164 accessions of wheat and related species; (2) assess the genetic differentiation and interspecific relationships among diploids, tetraploids, and hexaploids based on *Fst* values; (3) analyze the diversity and genetic differentiation in wheat and related species in order to understand the evolutionary pattern of *TaGW2* genes; (4) construct a *TaGW2* haplotype network that developed during polyploidization and track the haplotypes of *TaGW2-6A*, and -*6B* present in common wheat and known to have undergone strong selection; and 5) elucidate the relationship between *TaGW2* expression levels and grain weight in relation to polyploidization by real-time quantitative PCR. Finally, we also wished to compare the genetic diversity (π) and genetic differentiation of *TaGW2* promoters and coding regions among diploids, tetraploids, and hexaploids for a better understanding of wheat evolution using a key gene.

## Materials and methods

### Plant materials

The *TaGW2* sequences in 164 accessions of wheat and related species were generated. The accessions comprised 79 diploids, 55 tetraploids and 30 hexaploids (Table S1) and included 12 *T. urartu* (AA), 8 *T. boeoticum* (AA), 15 *T. monococcum* (AA), 12 *Ae. speltoides* (SS), 6 *Ae. longissima* (SS), 4 *Ae. sharonensis* (SS), 2 *Ae. searsii* (SS), 20 *Ae. tauschii* (DD), 8 *T. dicoccoides* (AABB), 14 *T. dicoccum* (AABB), 16 *T. durum* (AABB), 8 *T. turgidum* L. (AABB), 3 *T. carthlicum* (AABB), 2 *T. polonicum* (AABB), 2 *T. turanicum* (AABB), 2 *T. araraticum* (AAGG), and 30 *T. aestivum* (16 landraces and 14 modern cultivars, AABBDD) accessions. All were obtained from Chinese Crop Germplasm Resources Information System (http://www.cgris.net/zhongzhidinggou/index.php). Detailed information for each accession is given in Table S2.

### Phenotypic traits

Cultivars used in this study were planted at the CAAS-Shunyi Experiment Station in Beijing (116.3°E, 40.0°N) during the wheat-growing season. Each cultivar was planted in 2 m double rows spaced 25 cm apart, with 20 seeds planted in each row. Field management followed local practices. Mean widths (mm) of 20 kernels, and 100-grain weights of two samples for each accession converted to 1,000-kernel weight were obtained for analysis. Detailed information is provided in Table S3.

### DNA and RNA extraction

Genomic DNA was extracted from leaves of 15-day-old seedlings using the CTAB method (Chen and Ronald, 1999). Mature grains were ground to a powder in liquid nitrogen and total RNA was extracted using a TIANGEN RNAplant plus Reagent (Tiangen, Beijing) following instructions given with the kit. The cDNA was synthesized using the SuperScript II system (Invitrogen, Madison, WI, USA) according to the manufacturer's instructions, and then diluted 10-fold for subsequent quantitative real-time PCR (qRT-PCR) analysis.

### Primers and PCR amplification

Primers (Table S4) designed using Primer Premier 5.0 software (http://www.premierbiosoft.com/) was synthesized by Shanghai Sangon Biological Technology Co., Ltd (http://www.sangon.com/). PCR were performed in total volumes of 15 μL comprising 50 ng of genomic DNA, 1 μL of 10 mM forward and reverse primers, 0.24 μL of 25 mM dNTPs, 7.5 μL of GC Buffer I, and 0.15 μL of LA Taq Polymerase (Takara, Dalian). Samples for PCR were incubated at 94°C for 4 min, followed by 35 cycles of 94°C for 45 s, annealing for 45 s, and extension at 72°C for 30 s to 3 min, with a final extension for 10 min. The annealing temperature and extension time varied according to the primer set and size of PCR product (Table S4).

### Sequencing

Two pairs of primers (TaGW2-P-1 and TaGW2-P-2) for promoter amplification, and four pairs of primers (TaGW2-1, TaGW2-2, TaGW2-3, and TaGW2-4) for the coding sequences were designed for amplification (Table S4). PCR were as mentioned above. PCR products were separated by electrophoresis in agarose gels, and the target bands were extracted and cloned into pEASY-T1 simple vectors and transformed to DH5α-competent *Escherichia coli* by the heat shock method (Beijing Trans Gen Biotech Co., Ltd, Product Code: CT111). Positive clones were selected for sequencing by an ABI 3730XI DNA Analyzer (Applied Biosystems). PCR and DNA sequencing were repeated at least three times to ensure sequence accuracy. Promoter and coding sequences of *TaGW2s* for diploids, tetraploids, and hexaploids were submitted to GenBank (Accession numbers: BankIt1971968 KY264756-KY264772).

### Expression analysis

Genome-specific primers were designed according to cDNA sequence differences of the three homologous genes and used to evaluate the correlation of gene expression levels of *TaGW2-6A/6B/6D* and grain weight. The primer sets for *TaGW2* (*TaGW2-6A*-RT, *TaGW2-6B*-RT, and *TaGW2-6D*-RT) and Actin (Table S4) were used for amplification of *TaGW2* and actin genes, respectively. The qRT-PCR was conducted using mRNA extracted from mature seeds of diploids, tetraploids and hexaploids, with SYBR Premix Ex-Taq (Takara, Dalian) on a 7,500 Real-time PCR system (Applied Biosystems, Foster City, CA). qRT-PCR were performed in total volumes of 20 μL, containing 2 μL of cDNA, 1 μL of 2 mM gene-specific primers, 0.4 μL of ROX Reference Dye (50×), and 10 μL of 2×SYBR Premix Ex-Taq. The relative expression values of *TaGW2s* were calculated by the 2−ΔΔCt method using actin gene as endogenous control, which was not variable in different tissues and developmental stages of wheat under our experiment (Livak and Schmittgen, 2001; Bednarek et al., 2012). Each measurement was determined on at least two independent biological samples, with three replicates for each sample.

### Data analyses

The full length sequence alignments of *TaGW2-6A/6B/6D* genes were conducted using DNAStar (http://www.dnastar.com/). The *Fst* test was performed using Arlequin 3.5.1.2 (http://cmpg.unibe.ch/software/arlequin3/) based on the data from the software DNAStar. Diversity analyses, Tajima's *D*-tests (Tajima, 1989), and synonymous substitution tests were conducted using DnaSP 5.10 (http://www.ub.es/dnasp). Phylogenetic trees were drawn using MEGA 6.0 (http://www.megasoftware.net/). Haplotype networks based on the *TaGW2* DNA sequences were constructed using TCS 1.21 (Clement et al., 2000). Variance analyses and significance tests were performed using SPSS System for Windows Version 12.0 (http://www-01.ibm.com/software/analytics/spss/). Tukey's test was used to determine statistical differences by one-way ANOVA.

## Results

### Nucleotide polymorphism of *TaGW2s* in diploids, tetraploids, and hexaploids

**Figure 1:**
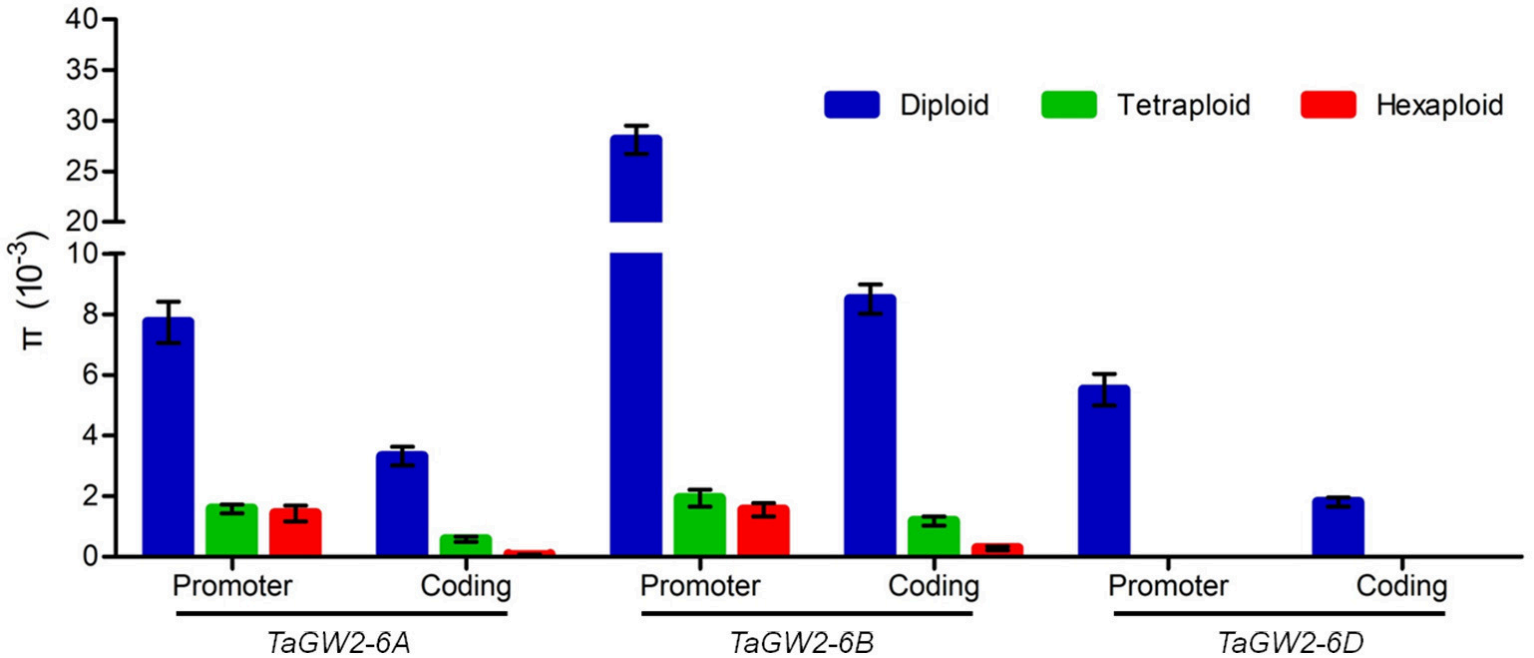
**Comparison of gene diversities (π) of ***TaGW2s*** between the promoter and coding regions in diploids (blue), tetraploids (green), and hexaploids (red)**. Bars represent the standard errors.

Nucleotide polymorphisms of the three *TaGW2* homoeologs were obtained from ~2.9 kb of promoter regions and ~8.8 kb of coding regions. Genetic diversity decreased significantly (*P* < 0.01) with progression from diploids to hexaploids (Table 1). For example, there were 119 polymorphisms (SNPs + InDels) in diploids (π = 4.64 × 10^−3^), 34 in tetraploids (π = 1.25 × 10^−3^), and only 10 in hexaploids (π = 0.60 × 10^−3^) at *TaGW2-6A*. At *TaGW2-6B*, the comparable frequencies were 284, 36 and 15 with π values of 15.51 × 10^−3^, 1.45 × 10^−3^, and 0.81 × 10^−3^, respectively. At *TaGW2-6D* the number of polymorphic sites was 93 in *Ae. tauschii* with none being identified in hexaploid wheat. Diversity in the promoter regions of *TaGW2s* was significantly higher than in the coding regions (*P* < 0.05), indicating a high level of conservation in the gene-coding regions (Table 1 and Figure 1). In both promoters and coding regions *TaGW2-6B* had the highest genetic diversity followed by *TaGW2-6A* and *TaGW2-6D* (Figure 1).

**Table 1:** **Nucleotide polymorphisms and π values of ***TaGW2*** genes in diploids, tetraploids, and hexaploids**.

**Gene**	**Region**	**Length (bp)**	**Diploids**				**Tetraploids**				**Hexaploids**			
			**SNPs**	**InDels**	**π ± S.E (10^−3^)**	***P*-value**	**SNPs**	**InDels**	**π ± S.E (10^−3^)**	***P*-value**	**SNPs**	**InDels**	**π ± S.E (10^−3^)**	***P*-value**
*TaGW2-6A*	Promoter	2400	37	3	7.74 ± 0.68a (A)	0.000	20	6	1.58 ± 0.15b (B)	0.016	7	2	1.43 ± 0.26c (C)	0.000
	Coding	8816	70	9	3.32 ± 0.31a (A)		7	1	0.58 ± 0.09b (B)		1	0	0.05 ± 0.02c (C)	
	Total	11216	107	12	4.64 ± 0.52a (A)		27	7	1.25 ± 0.24b (B)		8	2	0.60 ± 0.45c (C)	
*TaGW2-6B*	Promoter	2350	122	4	28.12 ± 1.39a (A)	0.000	15	5	1.93 ± 0.28b (B)	0.003	9	0	1.55 ± 0.22b (B)	0.000
	Coding	8888	150	8	8.50 ± 0.48a (A)		14	2	1.18 ± 0.15b (B)		4	2	0.28 ± 0.06c (C)	
	Total	11238	272	12	15.51 ± 0.82a (A)		29	7	1.45 ± 0.14b (B)		13	2	0.81 ± 0.11c (C)	
*TaGW2-6D*	Promoter	2400	30	5	5.51 ± 0.52a (A)	0.000	–	–	–		0	0	0 b (B)	
	Coding	8825	46	12	1.81 ± 0.15a (A)		–	–	–		0	0	0 b (B)	
	Total	11225	76	17	2.61 ± 0.34a (A)		–	–	–		0	0	0 b (B)	

*π, average number of nucleotide differences between two sequences. Capital and small letters indicate significance levels at P < 0.01 and P < 0.05 in comparison between ploidy levels for each region*.

**Figure 2:**
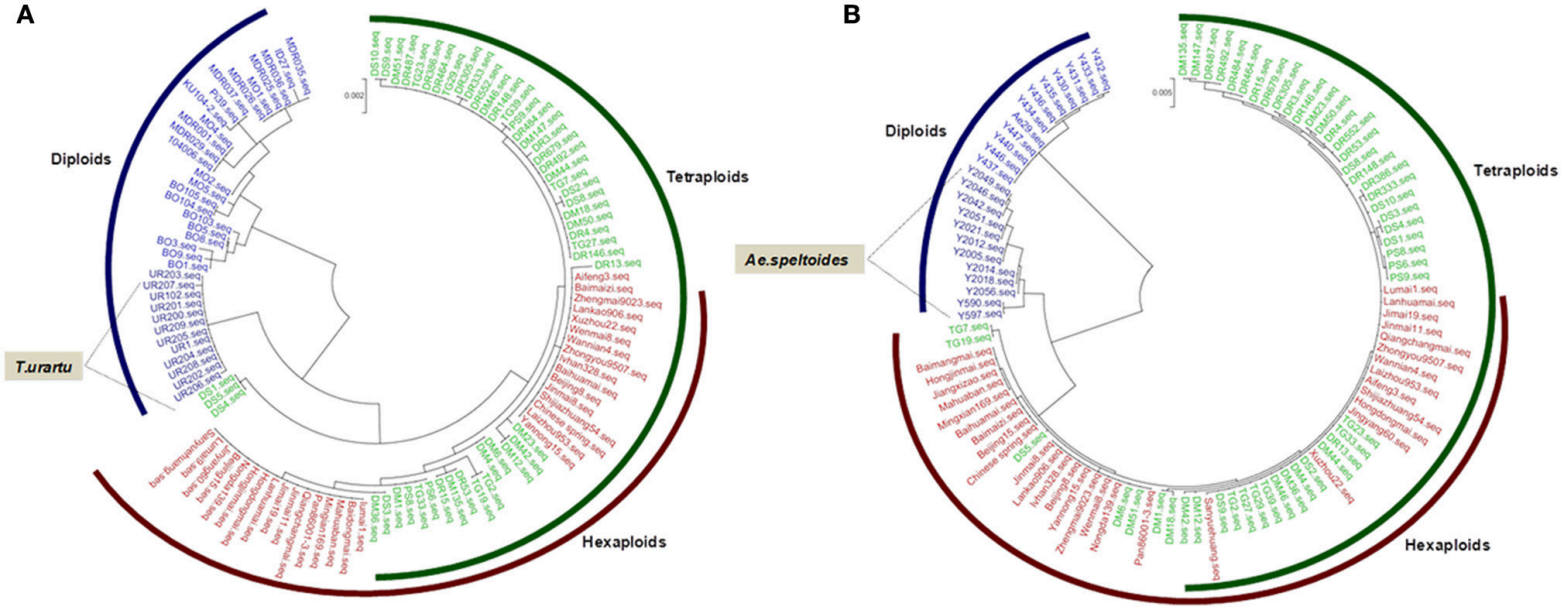
**Phylogenic analysis of ***TaGW2-6A*** (A)** and *TaGW2-6B* **(B)** in diploids (blue), tetraploids (green), and hexaploids (red).

Phylogenetic analysis of *TaGW2-6A* in wheat and its relatives showed that diploid *Triticum* species clustered into a single subgroup, but the boundary between tetraploid and hexaploid species was not distinct. Interestingly, *T. urartu* was more closely related to the hexaploids than *T*. *boeoticum* and *T. monococcum* indicating that it may be the direct donor of the A genome (Figure 2A). At *TaGW2-6B, Aegilops speltoides* accessions clustered into a single major subgroup, with tetraploid and hexaploid accessions placed in another major subgroup. The closest phylogenetic relationship involved *Ae. speltoides* leading us to hypothesize that this species is the direct donor, or a major contributor, of *TaGW2-6B* (Figure 2B). As predicted, common wheat and *Ae. tauschii* clustered into subgroups based on *TaGW2-6D* (Figure S1).

### The strongest genetic differentiation of *TaGW2s* occurred at polyploidization rather than during domestication or breeding

**Figure 3:**
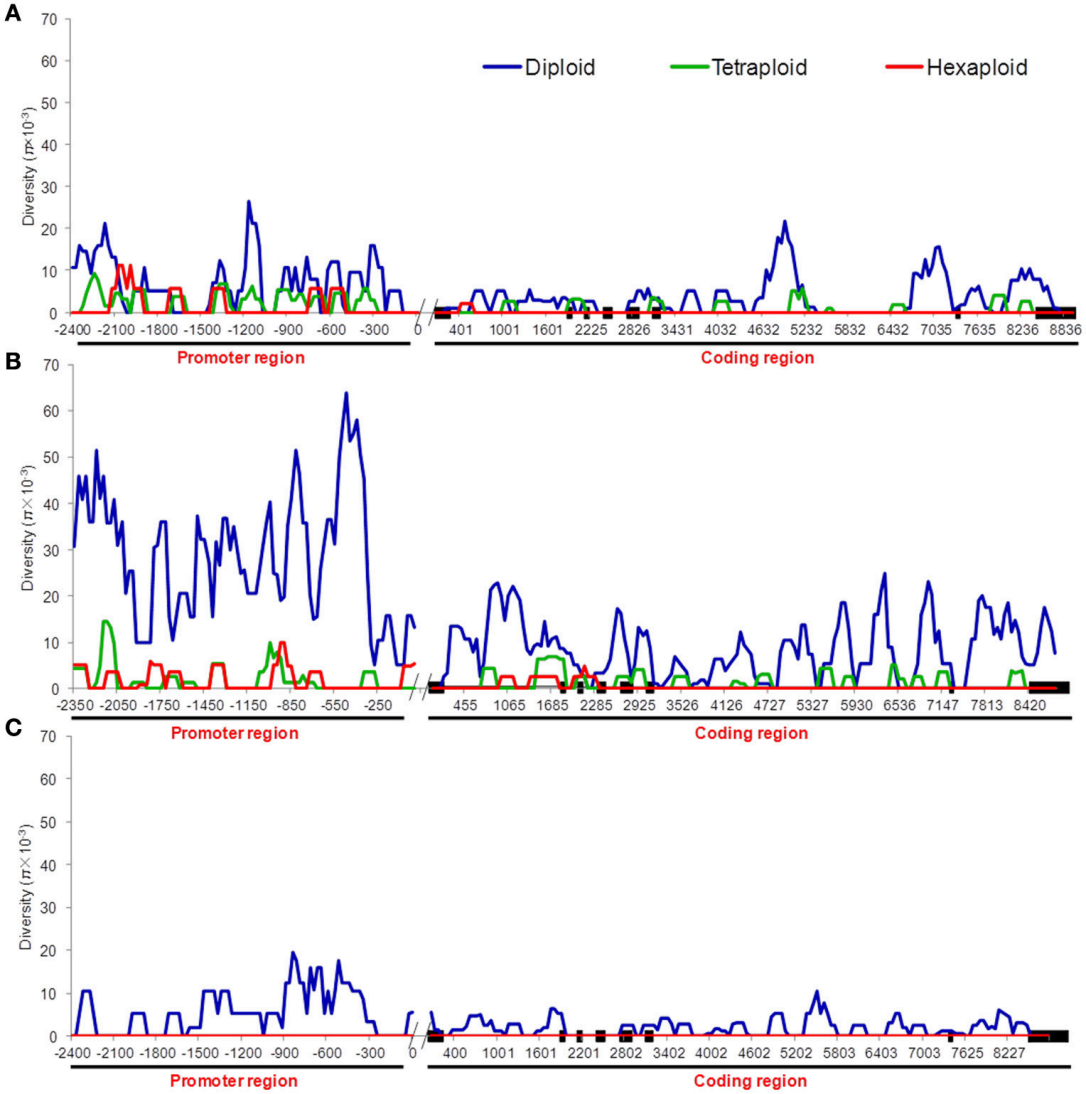
**π value comparison of ***TaGW2-6A*** (A)**, *TaGW2-6B* **(B)**, and *TaGW2-6D* **(C)** between promoter and coding regions in diploids (blue), tetraploids (green), and hexaploids (red). Black solid block in the horizontal axis indicates the exon, double slash indicates the boundary between coding and promoter regions, and numbers show the physical position of sequences.

**Figure 4:**
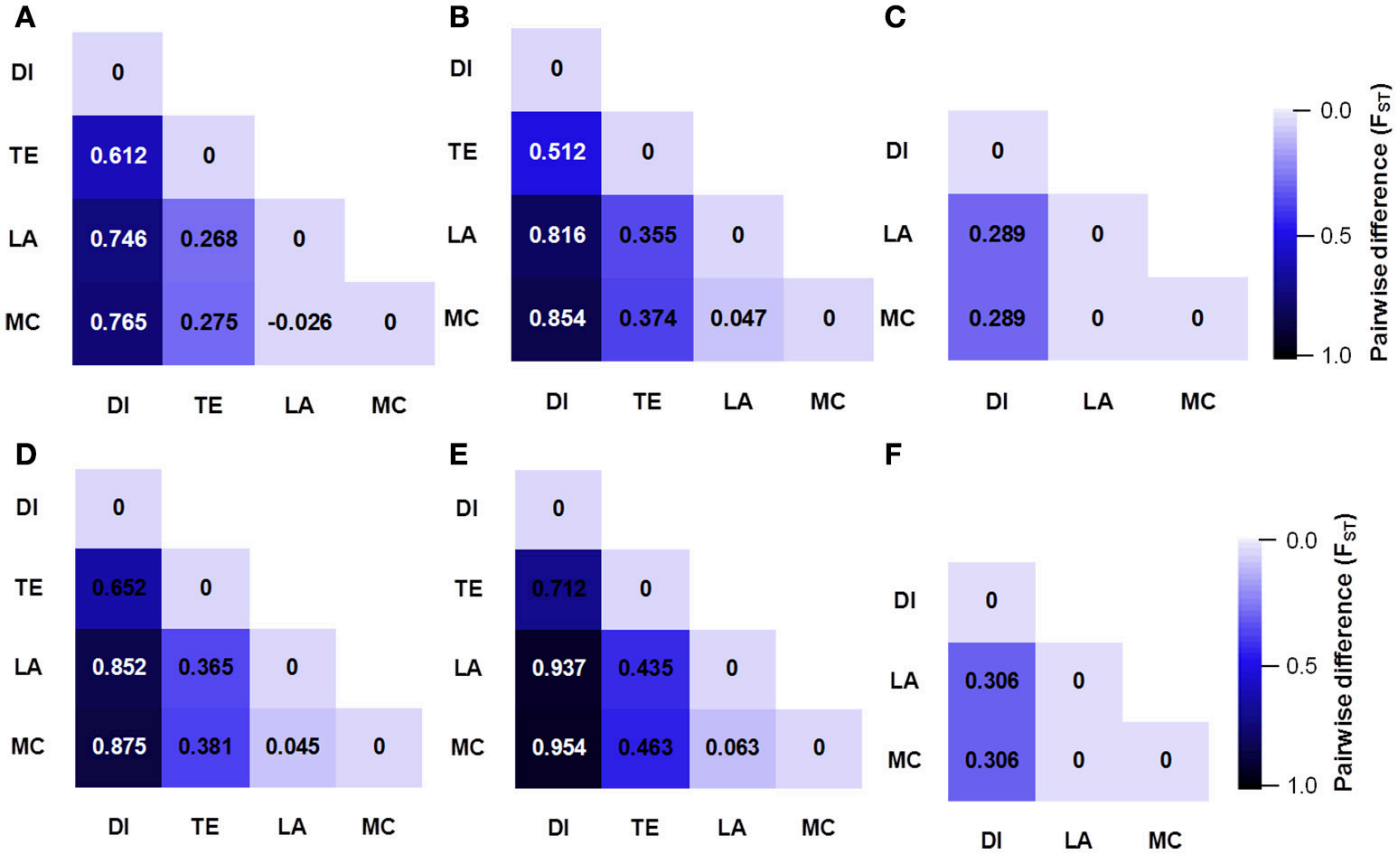
**Genetic differentiation (***Fst***) at coding and promoter regions of ***TaGW2-6A*** (A,D)**, *TaGW2-6B* **(B,E)**, and *TaGW2-6D* **(C,F)** between pairs of populations. The color gradient represents changes in *Fst* value from dark (1.0) to light blue (0.0). DI, diploids; TE, tetraploids; LA, landraces; MC, modern cultivars.

We compared the diversity and genetic differentiation of *TaGW2s* among hexaploids (modern cultivars and landraces), diploids, and tetraploids. A clear reduction in diversity occurred with progression from diploids to tetraploids (Figures 3, 4 and Table S5). *Fst* values for the coding and promoter regions of *TaGW2-6A* were 0.612 and 0.652 (*P* < 0.01) between diploids and tetraploids, 0.268 and 0.365 (*P* < 0.01) between tetraploids and landraces, and −0.026 and 0.045 between common wheat landraces and modern cultivars, respectively (Figure 4). In the coding region of *TaGW2-6B, Fst* was 0.512 (*P* < 0.01) between diploids and tetraploids, 0.355 and 0.374 (*P* < 0.01) between tetraploids and landraces and modern cultivars, and only 0.047 (*P* < 0.05) between landraces and modern cultivars. However, the *Fst* of the promoter region of *TaGW2-6B* was slightly higher than that of the coding region. In the coding region of *TaGW2-6D* the *Fst* between *Ae. tauschii* and common wheat was 0.289 (*P* < 0.01), and 0.306 in the promoter region (*P* < 0.01). There was no difference between landraces and modern cultivars in the coding and promoter regions of *TaGW2-6D* (Figures 3, 4). Therefore, the genetic differentiation between diploids and tetraploids was stronger than that between tetraploids and hexaploids in both the promoter and coding regions of *TaGW2s*. The *Fst* and π values (Figures 3, 4) also revealed stronger selection on *TaGW2* during tetraploidization than during hexaploidization. This was further supported by Tajima's tests. A significant deviation from the value of zero (*P* < 0.05) between the promoter and coding regions was detected in both diploids and hexaploids, thereby indicating that *TaGW2-6A* underwent strong selection at the regions of this locus (Table S6). Only the promoter region of *TaGW2-6B* in both diploids and hexaploids underwent selection, and *TaGW2-6D* underwent selection in hexaploids (Table S6).

Further comparison of polymorphisms and Tajima's *D*-values of *TaGW2s* in wheat and its relatives (Table 2) showed that the π values of the promoter and coding regions at *TaGW2-6A* were the highest in *T. boeoticum* (2.1 × 10^−3^ and 1.6 × 10^−3^). Tajima's *D*-values indicated that the promoter of *TaGW2-6A* underwent selection in both landraces and modern cultivars (*P* < 0.05), whereas in the coding region, selection occurred in *T. urartu* and *T. boeoticum*. The π values of the promoter and coding regions at *TaGW2-6B* were the highest in *Ae. speltoides* (4.82 × 10^−3^ and 1.98 × 10^−3^), and the coding region underwent strong selection (Table 2). According to Tajima's *D*-values, the strongest selection occurred in the promoter regions of both *TaGW2-6A* and *TaGW2-6B*.

**Table 2:** **Nucleotide polymorphisms and Tajima's ***D*** of ***TaGW2*** genes in wheat and related species**.

**Gene**	**Ploidy**	**Species**	**No. of accessions**	**Promoter**					**Gene**				
				**Length (bp)**	**SNPs**	**π (10^−3^)**	**θ (10^−3^)**	**Tajima's *D***	**Length (bp)**	**SNPs**	**π (10^−3^)**	**θ (10^−3^)**	**Tajima's *D***
*TaGW2-6A*	Diploid	*T. urartu*	12	2400	12	1.60	1.20	−1.45138	8816	30	1.16	1.14	0.07126^*^
		*T. boeoticum*	8		11	2.10	1.75	1.01793		41	1.60	1.78	−0.52090
		*T. monococcum*	14		13	1.78	1.65	0.32036		20	0.82	0.91	−0.40491
	Tetraploid	*T. dicoccoides*	8		12	2.23	1.89	0.90906		18	1.12	1.89	0.65742
		*T. dicoccum*	14		7	1.15	0.90	1.06282		12	0.58	0.90	0.73268
		*T. durum*	16		12	0.79	1.47	−1.77781		13	0.80	0.97	0.47695
		*T. turgidum*	8		8	1.31	1.25	0.20201		11	0.66	0.85	0.39853
	Hexaploid	Landraces	16		7	1.42	0.82	2.61475^**^		1	0.05	0.04	0.67135
		Modern cultivar	14		7	1.37	0.83	2.42088^*^		1	0.04	0.04	0.84865
*TaGW2-6B*	Diploid	*Ae. speltoides*	12	2350	30	4.82	4.21	0.64836	8888	39	1.98	1.82	1.38834^*^
		*Ae. longissima*	6		12	2.34	1.35	−0.05002		23	1.13	0.98	−0.43045
		*Ae. sharonensis*	4		15	2.66	2.87	−1.33698		28	1.07	1.00	0.31789
	Tetraploid	*T. dicoccoides*	8		9	1.62	1.39	0.79684		19	0.81	0.99	0.46725
		*T. dicoccum*	14		16	1.85	2.02	−0.33910		16	0.93	1.02	0.73653
		*T. durum*	16		14	1.86	1.70	0.38178		14	0.93	0.91	0.56473
		*T. turgidum*	8		8	1.38	1.24	0.53786		11	0.69	1.24	0.46378
	Hexaploid	Landraces	16		8	1.50	1.14	1.07627		5	0.27	0.19	1.71112
		Modern cultivar	14		9	1.60	1.02	1.44838		5	0.31	0.22	1.85358
*TaGW2-6D*	Diploid	*Ae. tauschii*	20	2400	30	5.46	3.39	0.86383	8825	46	1.81	1.50	0.86383
	Hexaploid	Landraces	16		0	0	0	—		0	0	—	—
		Modern cultivar	14		0	0	0	—		0	0	—	—

* Significant at P < 0.05;

** *Significant at P < 0.01*.

**Figure 5:**
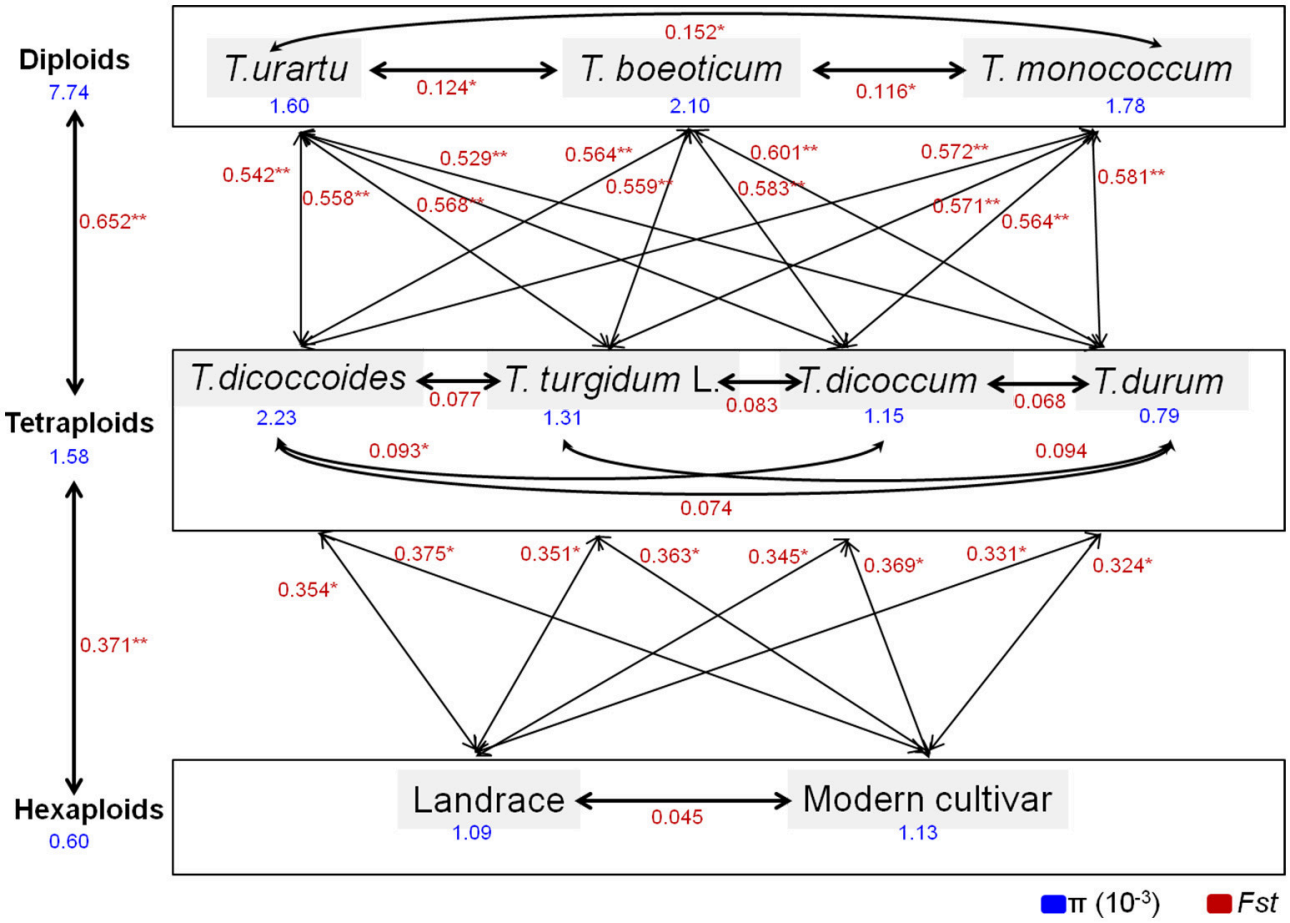
**π and ***Fst*** values for the promoter region of ***TaGW2-6A*** in wheat-related species**. Blue font indicates the value of genetic diversity (π), and red font shows the value of genetic differentiation (*Fst*). ^*^Significant at *P* < 0.05; ^**^Significant at *P* < 0.01.

Comparisons of nucleotide polymorphisms (π) and genetic differentiation (*Fst*) within and between ploidy levels in both the promoter and coding regions of *TaGW2-6A* (Figure 5 and Figure S2) showed that the π value in diploids was the highest, followed by the tetraploids, and lastly hexaploids. Genetic differentiation (*Fst*) in the diploids (*T. urartu, T. boeoticum*, and *T. monococcum*) varied from 0.10 to 0.16, in tetraploids (*T. dicoccoides, T. turgidum, T. dicoccum*, and *T. durum*) from 0.05 to 0.10, and in hexaploids (landrace and modern cultivar groups) less than 0.05. Compared to *TaGW2-6A* the π and *Fst* values for *TaGW2-6B* showed similar patterns of variation (Figures S3, S4). The *Fst* values among related wheat species also showed that stronger differentiation occurred at polyploidization rather than during domestication or breeding, and further indicated that the strongest selection occurred in the promoter regions of *TaGW2-6A* and *TaGW2-6B*.

### *TaGW2* haplotypes in common wheat can be traced back to tetraploid wheat groups

**Figure 6:**
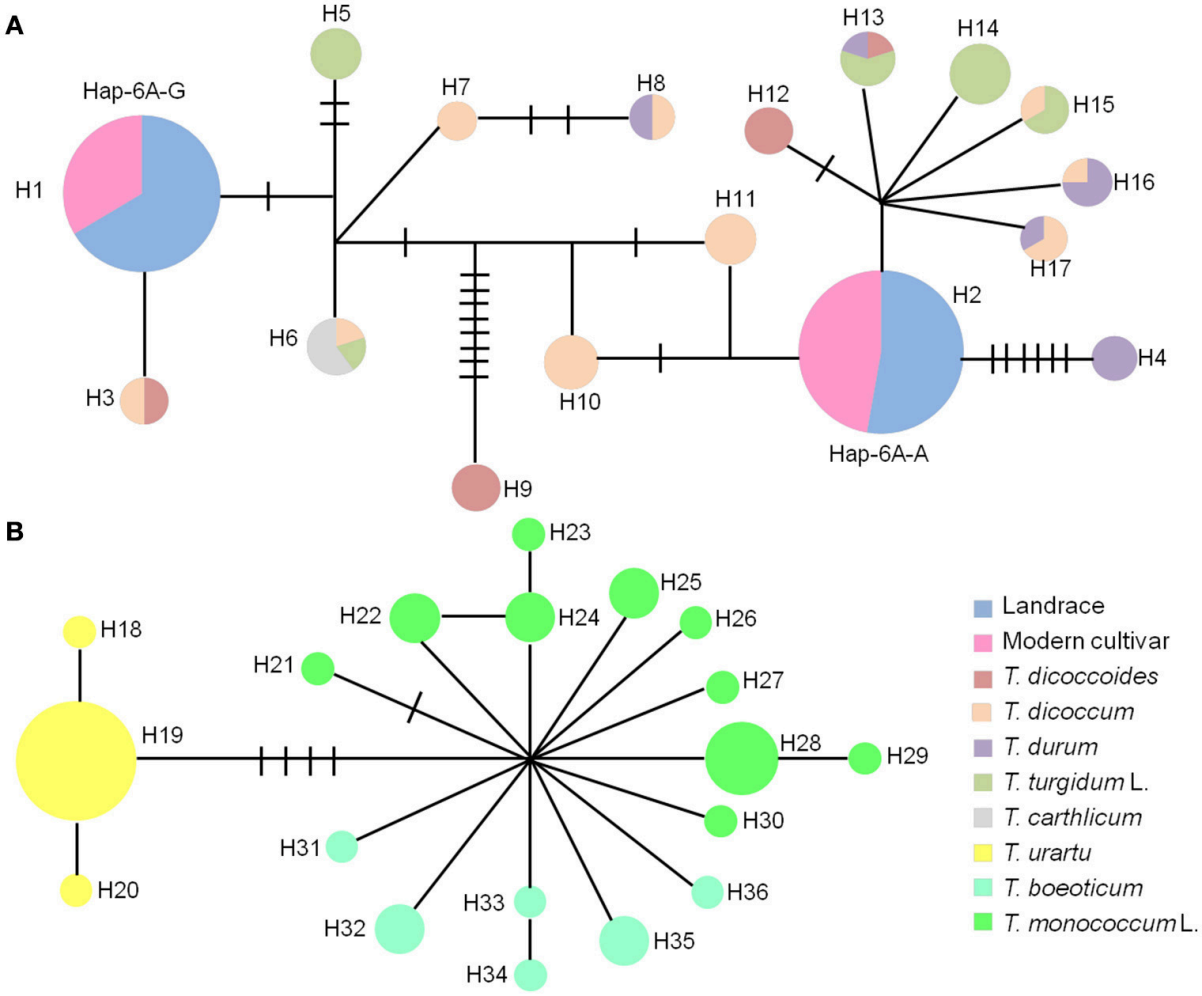
**Haplotype networks of ***TaGW2-6A*** based on promoter sequences in diploids, tetraploids and hexaploids. (A)** Haplotype networks of tetraploids and hexaploids. **(B)** Haplotype networks of diploids. Colored circles represent various subspecies.

The haplotype network in wheat relatives showed that the A genomes of *TaGW2* were clustered into two unconnected sub-networks. The hexaploids and tetraploids clustered in the same group, whereas diploids formed a distinct set (Figure 6). Fifteen *TaGW2-6A* haplotypes were detected in tetraploids and two in hexaploids, whereas there were 19 in diploids. The favorable (greater kernel width and weight) Hap-6A-A haplotype in common wheat was located close to that of *T. durum*, whereas Hap-6A-G was close to *T. dicoccoides* and *T. dicoccum*. The haplotype network of the B genomes also clustered into two sub-networks (Figure S5). The hexaploids and tetraploids clustered into the same sub-network, and diploids, including *Ae. speltoides, Ae. Longissima*, and *Ae. sharonensis*, were in a separate sub-network. Eleven haplotypes of *TaGW2-6B* were detected in tetraploids, whereas there were four in hexaploids, and 13 in the diploids. The favorable haplotype Hap-6B-1 detected in common wheat was located close to that of *T. dicoccoides* and *T. dicoccum*, whereas the origin of Hap-6B-2 was uncertain and could have been from any of the tetraploid species. The unfavorable haplotypes Hap-6B-3 and Hap-6B-4 formed a separate branch. The networks also showed the dramatic reduction in numbers of haplotypes at *TaGW2s* during polyploidization. A relative consistency of haplotypes existed between tetraploids and hexaploids.

**Figure 7:**
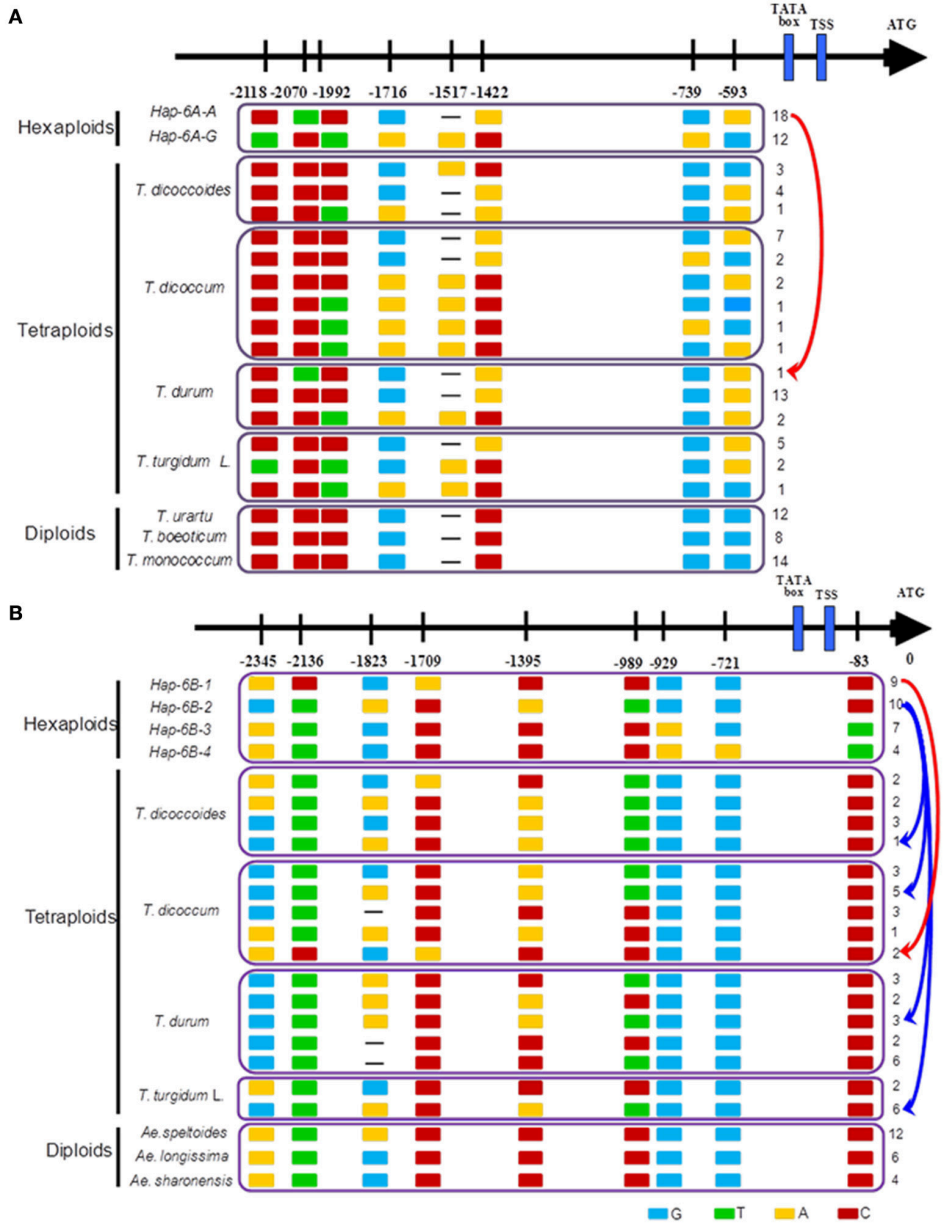
**Favored haplotypes of ***TaGW2-6A*** (A)** and *TaGW2-6B* **(B)** in hexaploids tracked to diploids and tetraploids. Rectangles colored brown, red, light blue and green represent nucleotide bases A, C, G, and T, respectively. Numbers indicate positions of polymorphism in promoters relative to the coding start codon.

Previous studies (Su et al., 2011; Qin et al., 2014) and haplotype network analysis in this study (Figure 7) detected two haplotypes in the promoter region of *TaGW2-6A*, and four haplotypes in *TaGW2-6B*. After tracking them through wheat polyploidization, we found that the favored haplotypes in *TaGW2-6A* and *TaGW2-6B* were all from tetraploids, i.e., Hap-6A-A was present in *T. durum* (DR13), Hap-6B-1 in *T. dicoccum* (DM6 and DM51), and Hap-6B-2 in *T. dicoccoides* (DS10), *T. dicoccum* (DM4, DM44, DM46, DM135, and DM147), *T. durum* (DR13, DR487, and DR492), and *T. turgidum* L. (TG2, TG23, TG27, TG29, TG33, and TG39). More importantly, SNPs discovered in hexaploids were almost all monomorphic in diploids, but polymorphic in tetraploids.

### *TaGW2s* negatively regulate seed size

**Figure 8:**
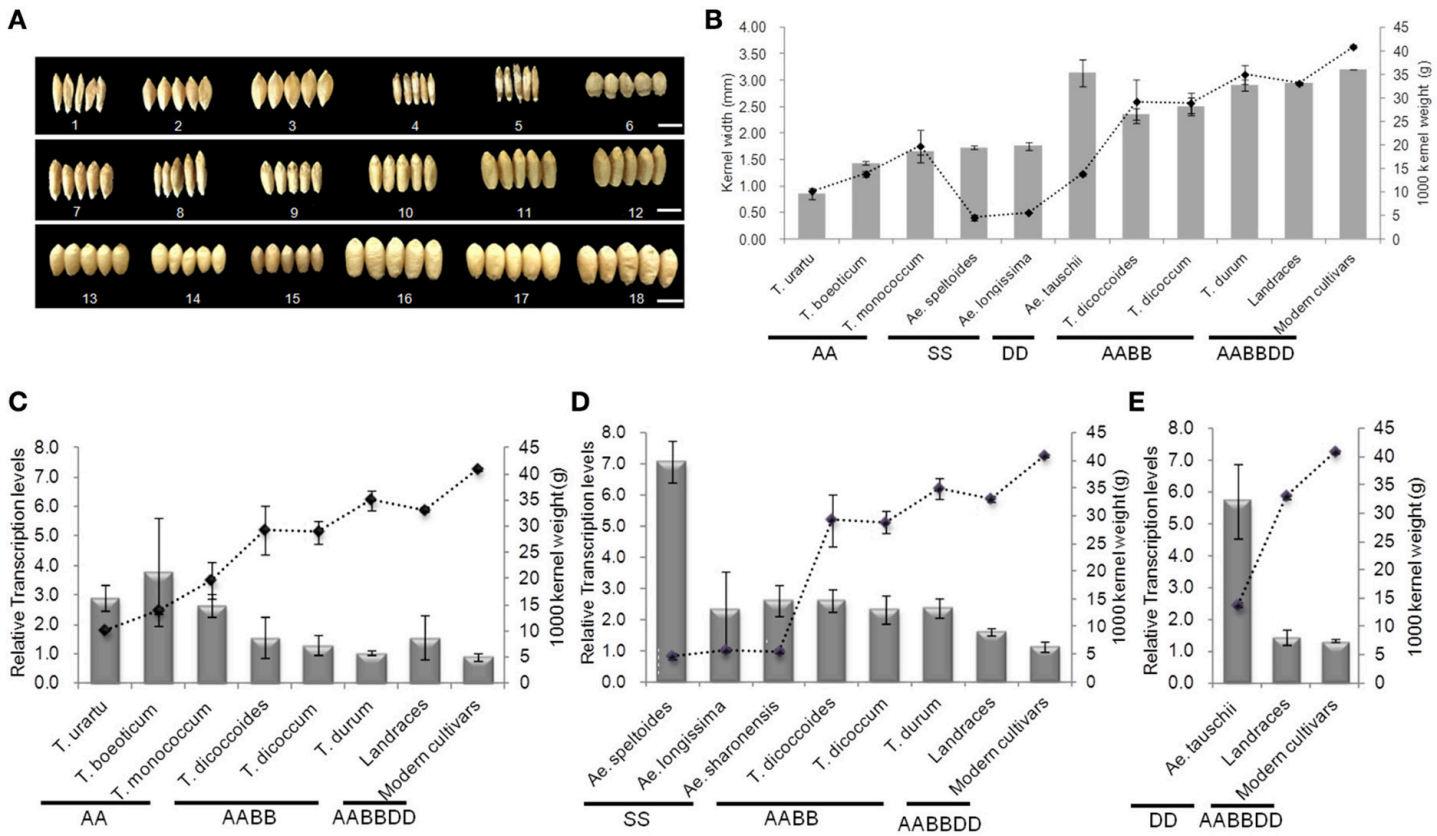
**Relationships between mean relative expression levels of ***TaGW2s*** and 1000-kernel weight in diploids, tetraploids, and hexaploids. (A)** Kernel shape, 1–6: diploids; 7-12: tetraploids; 13-18: hexaploids; bars, 5 mm. (A) 1: UR207 (AA); 2: BO8 (AA); 3: MO1 (AA); 4: Y590 (SS); 5: Y435 (SS); 6: Y2280 (DD); 7: DS4; 8: DS8; 9: DM12; 10: DM147; 11: DR3; 12: DR146; 13: Baihuamai; 14: Chinese Spring; 15: Nongda 139; 16: Zhongyou 9507; 17: Wenmai 8; 18: Zhengmai 9023. **(B)** Kernel weight and width. Bars represent standard errors. **(C–E)** Mean relative expression levels of *TaGW2-6A, TaGW2-6B*, and *TaGW2-6D*.

Genome-specific primers were designed according to cDNA sequence differences in the *GW2* homologs on chromosomes 6A, 6B, and 6D, in order to evaluate the correlation between respective gene expression levels and grain width/weight during wheat polyploidization (Figure 8 and Table S7). Grain width and grain weight obviously increased following polyploidization (Figures 8A,B). The average relative expression of *TaGW2-6A* decreased from 3.128 in diploids to 1.281 in tetraploids, and 1.148 in hexaploids, whereas the average grain weights and widths increased from 14.560 g and 1.552 mm to 31.824 g and 2.603 mm in tetraploid, and 35.846 g and 3.155 mm in common wheat, respectively (Figure 8C and Table S7). The differences in expression levels were not significant. The average relative expression of *TaGW2-6B* in diploids was 5.168, in tetraploids 2.426, and in hexaploids 1.434 (Figure 8D and Table S7). The decreased expression levels were significant *(P* < 0.05), as were the increases in grain width and weight (*P* < 0.01). Similar results were obtained for *TaGW2-6D* (*P* < 0.05) (Figure 8E and Table S7). In addition, we measured overall relative expression values of *TaGW2s* in diploids, tetraploids and hexaploids (Table S8). The overall transcription levels of *TaGW2-6A/6B/6D* were 3.128, 5.168, and 5.734 in diploids, respectively. The overall relative expressions of *TaGW2-6B/6D* were significantly higher than that in tetraploids (3.426, *P* < 0.05) and in hexaploids (3.530, *P* < 0.05), as grain width and weight increased, which reflected that expression level for each genome was dramatically declined in wheat polyploidization. This further demonstrated the negative regulatory roles of *TaGW2s* on grain size, and strong selection of these yield-related genes during wheat evolution.

## Discussion

### *TaGW2s* underwent stronger differentiation during wheat polyploidization than domestication and breeding

Common wheat has undergone ~8,000 years of artificial selection (Doebley et al., 2006; Feldman et al., 2012; Marcussen et al., 2014). The process of polyploidization of wheat involved a strong differentiation compared to the wild ancestral species, and genetic diversity significantly decreased, especially genes controlling important agronomic traits (Haudry et al., 2007).

In the present study, we compared sequence differences in *TaGW2* homologs in diploids, tetraploids and hexaploid wheat species and various relatives. Dramatic declines in nucleotide diversity (π) and *Fst* values (Figures 1, 3, 4 and Table 1) occurred with each round of polyploidization. As shown in Figure 5 and Figure S2 π values for the promoter and coding regions of *TaGW2-6A* decreased 4.8- and 5.7-fold, respectively, from diploids to tetraploids, and further decreased 2.6- and 11.6-fold from tetraploids to hexaploids. For *TaGW2-6B* comparable 14- and 1.23-fold reductions occurred at the promoter and coding regions with tetraploidy, and further reductions of 7.2 and 4.2 times occurred with hexaploidy (Figures S3, S4). However, π value differences among accessions within ploidy levels varied by less than 1-fold. Moreover, *Fst* values of *TaGW2-6A, -6B*, and *-6D* in both promoter and coding regions between diploids and tetraploids were higher than between tetraploids and hexaploids (Figure 4). In addition, the haplotype numbers of *TaGW2-6A* and *TaGW2-6B* decreased from diploids, to tetraploids and hexaploids (Figure 6 and Figure S5). Dramatic reductions in diversity in other genes, such as *TaSUS1-7A, TaGS5-5A*, and *TaCWI*, following polyploidization reported in other species (Hou et al., 2014; Jiang et al., 2015; Ma et al., 2016). All of these reports indicate that strong differentiation of important yield-related genes occurred during polyploidization and domestication of both tetraploid and hexaploid wheats.

### *T. urartu* and *Ae. speltoides* confirmed as the direct donors of the wheat A and B genomes

Common wheat arose following chromosome doubling of a natural hybrid of tetraploid *T. dicoccum* and diploid *Ae. tauschii*. This event that may have occurred as few as once or twice times caused an “evolutionary bottleneck”, and consequently much of the genetic variation present in diploid species and tetraploids with common genomes is not present in the hexaploid (Ogbonnaya et al., 2005; Ozkan et al., 2005).

In this study, the genetic relationships of common wheat and related species (Figure 2 and Figure S1) were evaluated by phylogenetic analysis of *TaGW2* polymorphisms. In regard to *TaGW2-6A* and *TaGW2-6B* tetraploids and hexaploids were in a single subgroup, with diploid species individually clustered into different subgroups, consistent with earlier results of Buckler et al. (2001). During the evolution of common wheat, *T. urartu* and a B genome donor (herein suggested to be *Ae. speltoides*) hybridized to form tetraploid wheat, which later hybridized with the D genome donor *Ae. tauschii* to form common wheat. Many studies have focused on the prospective A, B, D genome donors of wheat (Kihara, 1944; McFadden and Sears, 1944; Dvorák et al., 1993; Kilian et al., 2007). *T. urartu* (Dvorák et al., 1993), *Ae. speltoides* (Petersen et al., 2006; Kilian et al., 2007) and *Ae. tauschii* (Kihara, 1944) may be the direct or main donors of the A, B and D genomes, respectively. In this study, phylogenetic analysis of *TaGW2s* further verified *T. urartu* as the direct donor of the A genome, *Ae. speltoides* was the likely donor or main donor of the B genome, and *Ae. tauschii* was the D genome donor of common wheat (Figure 2 and Figure S1).

### Diversity differences in *TaGW2s* mainly occurred in the promoter regions during polyploidization of wheat

Natural diversity influencing gene expression levels of some yield-related genes in graminaceous crops often occurs in the promoter regions. Examples include *OsGS5, ZmGS3, ZmGW2-CHR4, TaTEF-7A*, and *TaCWI-4A* (Li et al., 2010a,b, 2011; Zheng et al., 2014; Jiang et al., 2015). Previous studies (Su et al., 2011; Qin et al., 2014) also showed that genetic diversity mainly occurred in the promoter regions of *TaGW2-6A* and *TaGW2-6B* in common wheat. Expression levels of *TaGW2* genes in developing seeds were negatively correlated with grain width and grain weight.

In the present study diversity (π) in the promoter regions was significantly higher than in the coding regions of *TaGW2* genes in various species (Table 1 and Figure 1). Genetic differentiation (*Fst*) in the promoter regions was higher than in the coding regions among diploids, tetraploids, and hexaploid groups (Figure 4). In addition, deviations of Tajima's *D* from zero for *TaGW2-6A* and *-6B* in the promoter regions of diploids and hexaploids further demonstrated that the promoter regions underwent selection. Compared to the conserved coding regions, the extensive variation that occurred in the promoter regions regulated grain size through variation in expression level. Correlation of gene expression levels of *TaGW2-6A, -6B* and *-6D* with grain width/weight during wheat polyploidization showed that grain width/weight increased with progression from diploids to hexaploids, but the relative expression levels of the genes significantly decreased (Figure 8 and Table S7).

### *TaGW2s* are conserved in function but have different fates in rice and wheat

*OsGW2*, first cloned in rice following genetic analysis of an induced mutant, encodes a ubiquitin E3 ligase (Song et al., 2007). There was no significant variation between landraces and modern cultivars, indicating that the locus had not been subjected to selection during domestication and breeding. Moreover, *indica* and *japonica* sub-populations showed different patterns of variation, suggesting that *OsGW2* might have undergone long-term purifying selection during evolution and improvement of rice (Lu et al., 2013). Huang et al. (2012) performed a genome-wide association study (GWAS) of flowering time and grain yield traits in a panel of 950 worldwide rice varieties and did not detect an association of *OsGW2* and yield. *TaGW2s* in wheat are functional RING-type E3 ligases (Bednarek et al., 2012), and gene expression analysis and RNAi demonstrated that variation in them was negatively correlated with grain weight, a function that was similar to *OsGW2* in rice (Su et al., 2011; Yang et al., 2012; Hong et al., 2014; Qin et al., 2014). Strong selection of certain *TaGW2-6A* and *TaGW2-6B* haplotypes occurred in global wheat breeding (Su et al., 2011; Qin et al., 2014). In this study, we conducted a systematic analysis of the *TaGW2* genes during polyploidization of wheat. Haplotype networks and haplotype analyses (Figures 6, 7 and Figure S5) showed that favorable haplotypes of *TaGW2-6A* and *TaGW2-6B* in common wheat were also found in tetraploids, but were not detected in diploids. Strong selection of favorable variants of *TaGW2-6A* and *TaGW2-6B* apparently occurred in both tetraploid and hexaploid wheats. Clearly the agronomic effects of variation in *TaGW2* genes in polyploid wheat and rice were different. This work demonstrates the value of comparative gene homology studies in grass species.

## Author contributions

LQ, CH, and XZ designed research. LQ, JZ, and CH performed research. LQ, TL, and JH contributed new reagents or analytical tools. LQ and CH analyzed data. LQ, XZ, and CH drafted the manuscript.

### Conflict of interest statement

The authors declare that the research was conducted in the absence of any commercial or financial relationships that could be construed as a potential conflict of interest.
