# Supplementary material for: TaGW2, a Good Reflection of Wheat Polyploidization and Evolution
Source: Front Plant Sci. 2017 Mar 7;8:318. doi: 10.3389/fpls.2017.00318 (PMC5339256; doi:10.3389/fpls.2017.00318)
Supplement: Supplementary file 1 [file DataSheet1.PDF]

Supplementary Material

TaGW2, a good reflection of wheat polyploidization and evolution

Lin Qin[1,2], Junjie Zhao[2], Tian Li[2], Jian Hou[2], Xueyong Zhang[1,2*], and Chenyang Hao[2*]

Supplementary Figures and Tables

Supplementary Figure 1. Phylogenic analysis of *TaGW2-6D* in diploids (blue) and hexaploids (red).

Supplementary Figure 2. *π* and *Fst* values for the coding region of *TaGW2-6A* in wheat-related species. Blue font indicates the value of genetic diversity (*π*), and red font shows the value of genetic differentiation (*Fst*). * significant at *P*<0.05; ** significant at *P*<0.01.

Supplementary Figure 3. *π* and *Fst* values for the promoter region of *TaGW2-6B* in wheat-related species. Blue font indicates the value of genetic diversity (*π*), and red font shows the value of genetic differentiation (*Fst*). * significant at *P*<0.05; ** significant at *P*<0.01.

Supplementary Figure 4. *π* and *Fst* values for the coding region of *TaGW2-6B* in wheat-related species. Blue font indicates the value of genetic diversity (*π*), and red font shows the value of genetic differentiation (*Fst*). * significant at *P*<0.05; ** significant at *P*<0.01.

Supplementary Figure 5. Haplotype networks of *TaGW2-6B* based on promoter sequences in diploids, tetraploids and hexaploids. (A) haplotype networks of tetraploids and hexaploids. (B) haplotype networks of diploids. Colored circles represent various subspecies.

Supplementary Table 1. Materials used for sequencing *TaGW2*s in this study.

Supplementary Table 2. Detailed information for accessions of wheat and related species used in this study.

Supplementary Table 3. Kernel width (KW) and 1000-kernel weight (TKW) of wheat relative species.

Supplementary Table 4. Primer sequences used in this study.

Supplementary Table 5. *P* value of each *Fst* value for coding and promoter regions of *TaGW2-6A* (A, D), *TaGW2-6B* (B, E) and *TaGW2-6D* (C, F) between different populations.

Supplementary Table 6. Tajima's *D* evaluations of promoter and coding regions of *TaGW2* genes in diploid, tetraploid and hexaploid accessions.

Supplementary Table 7. Mean relative expressions level of *TaGW2* genes, kernel width and 1000-kernel weight in wheat and related species.

Supplementary Table 8. Overall relative expressions level of *TaGW2* genes, kernel width and 1000-kernel weight in diploids, tetraploids and hexaploids.

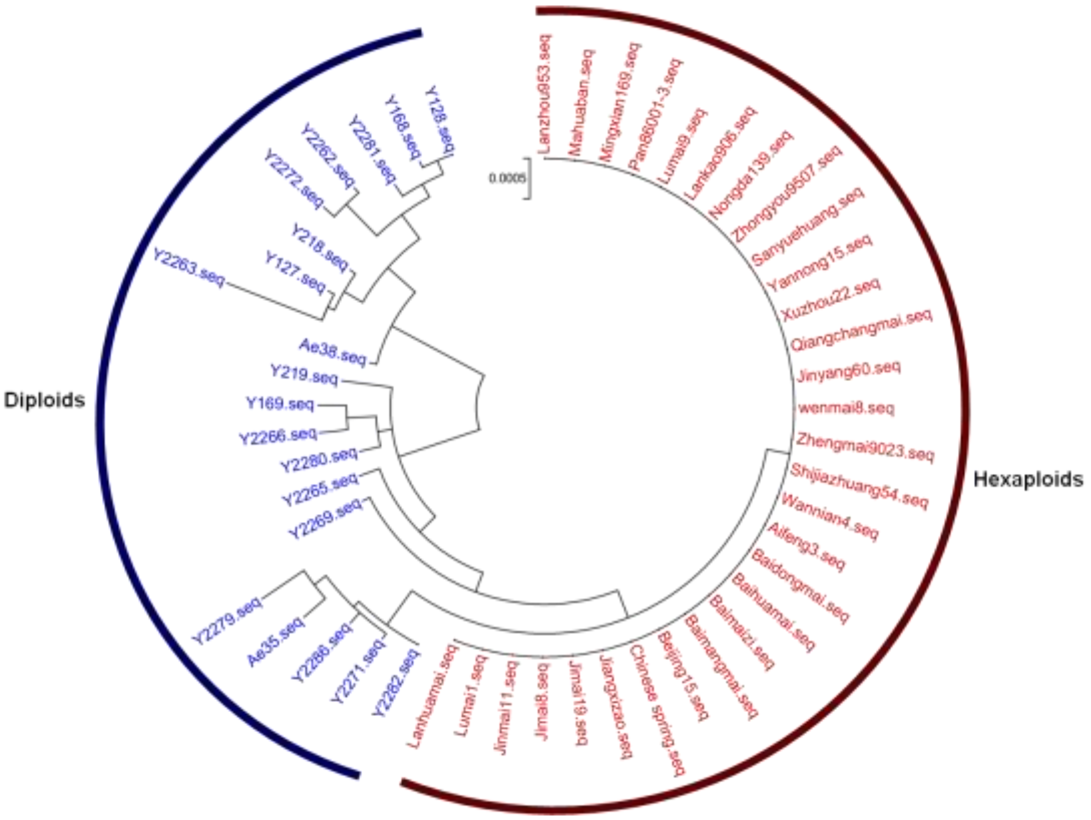

Diploids
Hexaploids
0.0005
Y128.seq
Y168.seq
Y2281.seq
Y2262.seq
Y2272.seq
Y218.seq
Y127.seq
Y2263.seq
Ae38.seq
Y219.seq
Y169.seq
Y2266.seq
Y2280.seq
Y2265.seq
Y2269.seq
Y2279.seq
Ae35.seq
Y2286.seq
Y2271.seq
Y2282.seq
Lanhuamai.seq
Lumai1.seq
Jinmai11.seq
Jimai8.seq
Jimai19.seq
Jiangxizao.seq
Chinese spring.seq
Beijing15.seq
Baimangmai.seq
Baimaizi.seq
Baihuamai.seq
Baidongmai.seq
Aifeng3.seq
Wannian4.seq
Shijiazhuang54.seq
Zhengmai9023.seq
wenmai8.seq
Jinyang60.seq
Qiangchangmai.seq
Xuzhou22.seq
Yannong15.seq
Sanyuehuang.seq
Zhongyou9507.seq
Nongda139.seq
Lankao906.seq
Lumai9.seq
Pan86001-3.seq
Mingxian169.seq
Mahuaban.seq
Lanzhou953.seq

Figure S1

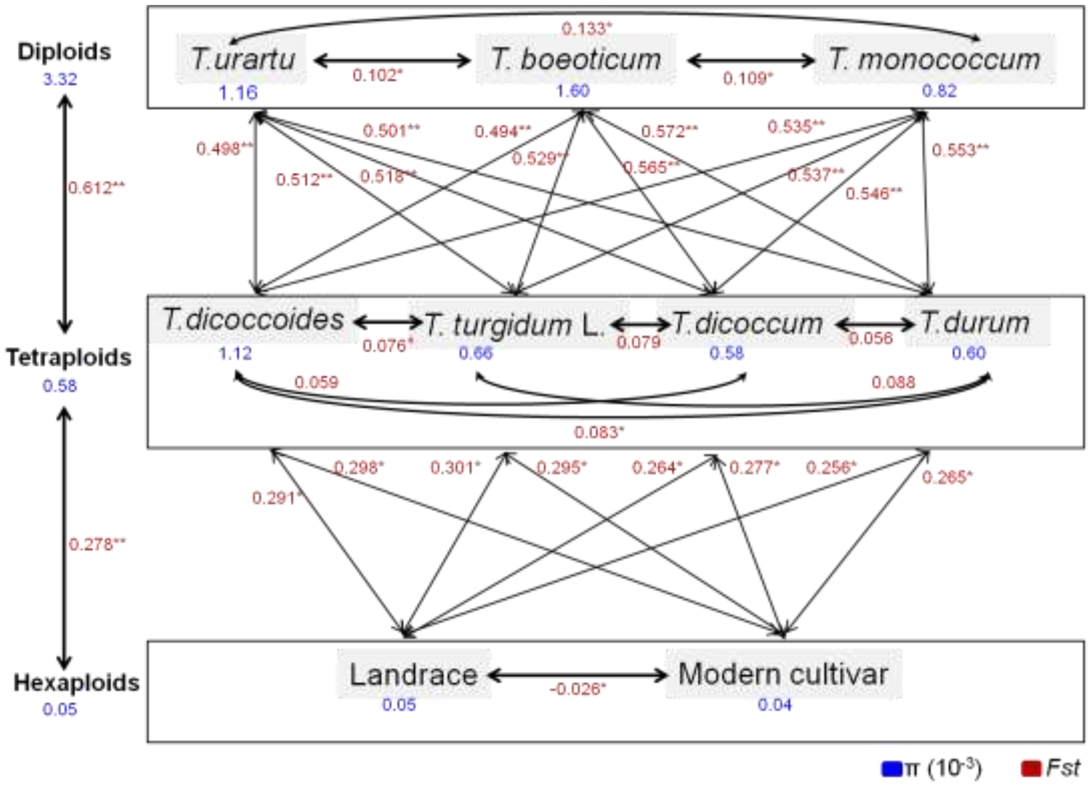

Diploids
3.32
Tetraploids
0.58
Hexaploids
0.05
0.612**
0.278**
T.urartu
1.16
T. boeoticum
1.60
T. monococcum
0.82
0.133*
0.102*
0.109*
0.498**
0.501**
0.494**
0.572**
0.535**
0.553**
0.529**
0.565**
0.537**
0.512**
0.518**
0.546**
T.dicoccoides
1.12
T. turgidum L.
0.66
T.dicoccum
0.58
T.durum
0.60
0.076*
0.079
0.056
0.059
0.088
0.083*
0.298*
0.301*
0.295*
0.264*
0.277*
0.256*
0.265*
0.291*
Landrace
0.05
Modern cultivar
0.04
-0.026*
π (10-3)
Fst

Figure S2

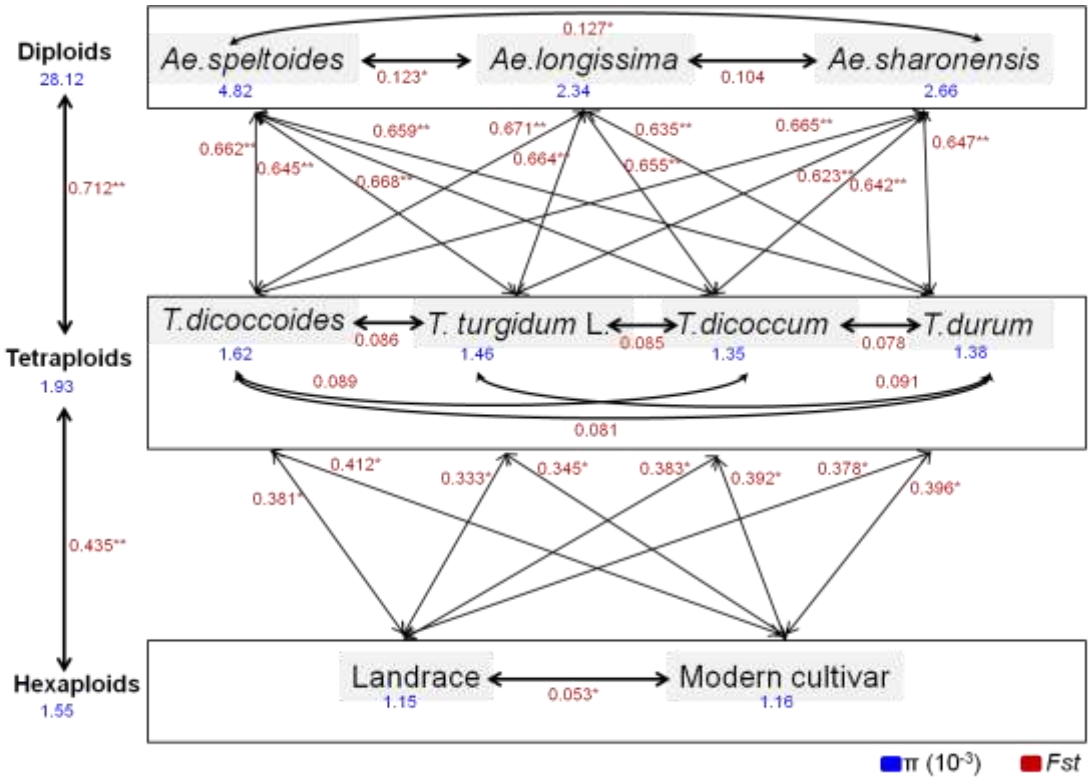

Diploids
28.12
Ae.speltoides
4.82
Ae.longissima
2.34
Ae.sharonensis
2.66
0.127*
0.123*
0.104
0.712**
0.662**
0.645**
0.659**
0.668**
0.671**
0.664**
0.635**
0.655**
0.665**
0.623**
0.642**
0.647**
Tetraploids
1.93
T.dicoccoides
1.62
T. turgidum L.
1.46
T.dicoccum
1.35
T.durum
1.38
0.086
0.085
0.078
0.089
0.091
0.081
0.435**
0.412*
0.381*
0.333*
0.345*
0.383*
0.392*
0.378*
0.396*
Hexaploids
1.55
Landrace
1.15
Modern cultivar
1.16
0.053*
π (10-3)
Fst

Figure S3

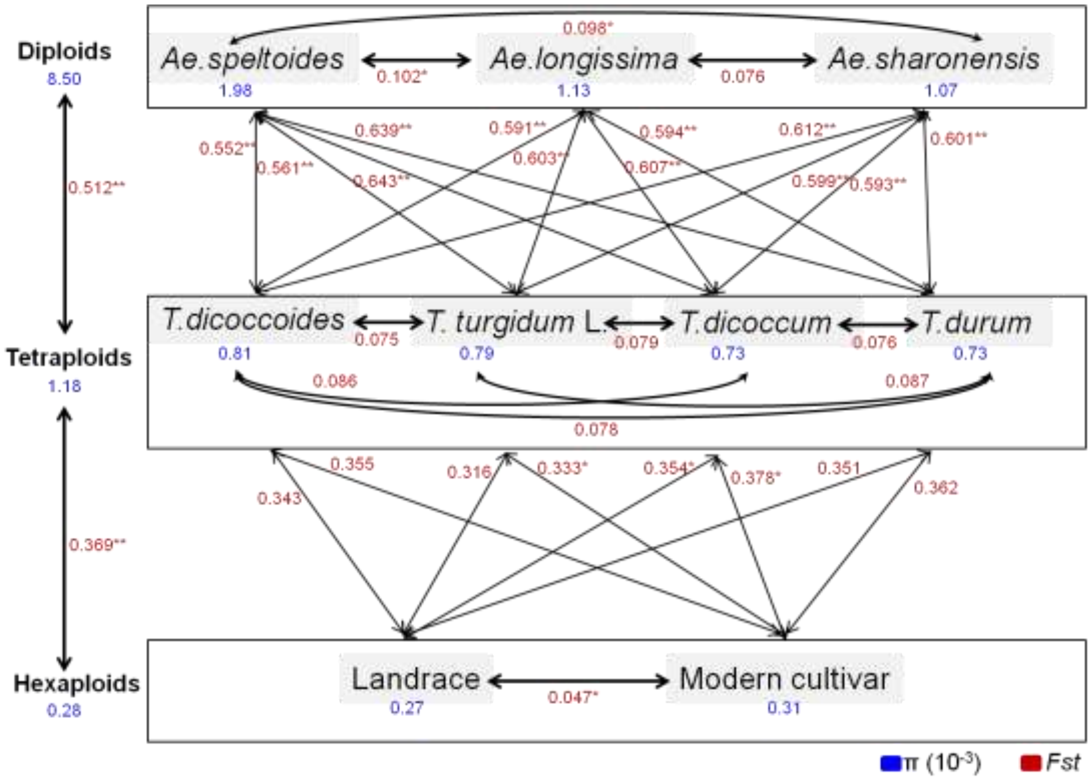

Diploids
8.50
Ae.speltoides
1.98
Ae.longissima
1.13
Ae.sharonensis
1.07
0.098*
0.102*
0.076
0.512**
0.552**
0.561**
0.639**
0.643**
0.591**
0.603**
0.594**
0.607**
0.612**
0.599**
0.593**
0.601**
Tetraploids
1.18
T.dicoccoides
0.81
T. turgidum L.
0.79
T.dicoccum
0.73
T.durum
0.73
0.075
0.079
0.076
0.086
0.087
0.078
0.369**
0.355
0.343
0.316
0.333*
0.354*
0.378*
0.351
0.362
Hexaploids
0.28
Landrace
0.27
Modern cultivar
0.31
0.047*
π (10-3)
Fst

Figure S4

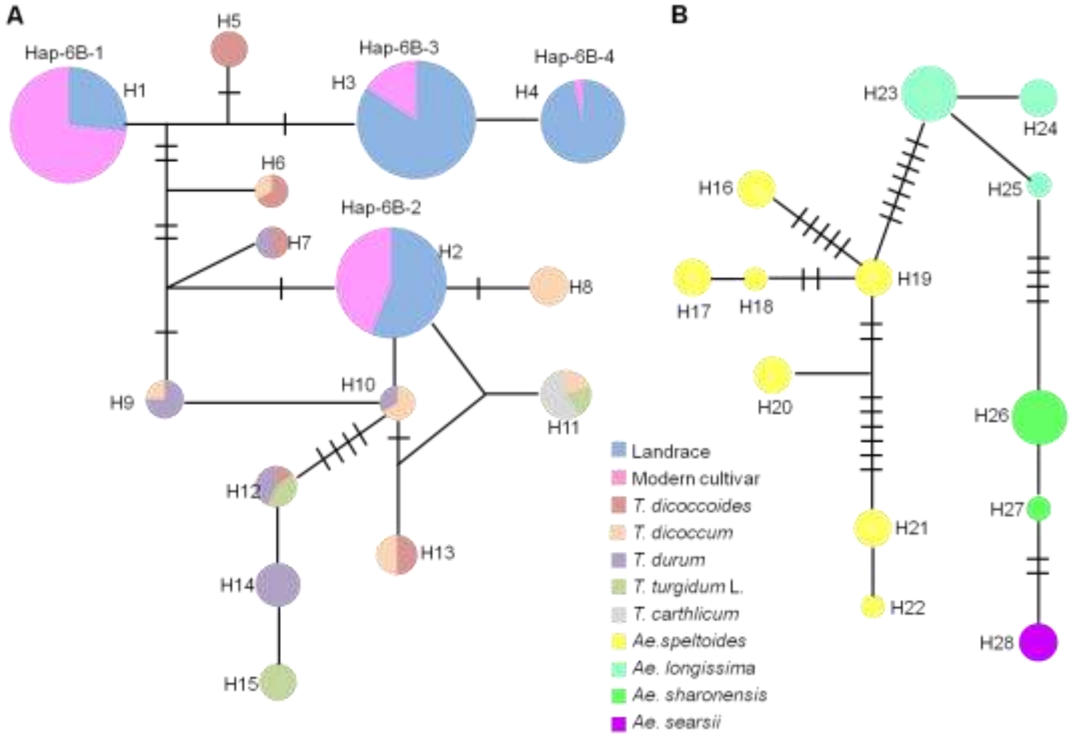
A
B
Hap-6B-1
H1
H5
Hap-6B-3
H3
Hap-6B-4
H4
H6
Hap-6B-2
H7
H2
H8
H9
H10
H11
H12
H13
H14
H15
Landrace
Modern cultivar
T. dicoccoides
T. dicoccum
T. durum
T. turgidum L.
T. carthlicum
Ae.speltoides
Ae. longissima
Ae. sharonensis
Ae. searsii
H23
H24
H25
H16
H19
H17
H18
H20
H26
H27
H21
H22
H28

Figure S5

Table S1

Ploidy	Species	Genome	No. of accessions	Total
Diploids	*T. urartu*	A^u	12	79
	T. boeoticum	A^b	8	
	T. monococcum L.	A^m	15	
	Ae. speltoides	S	12	
	Ae. longissima	S^l	6	
	Ae. sharonensis	S^{sh}	4	
	Ae. searsii	S^s	2	
	Ae. tauschii	D	20	
Tetraploids	*T. dicoccoides*	AB	8	55
	T. dicoccum	AB	14	
	T. durum	AB	16	
	T. turgidum L.	AB	8	
	T. carthlicum	AB	3	
	T. polonicum	AB	2	
	T. turanicum	AB	2	
	T. araraticum	AB	2	
Hexaploids	*T. aestivum*_landraces	ABD	16	30
	*T. aestivum*_modern cultivars	ABD	14	
	Total			164

Table S2

Code	Species	Accession	Genome	Variety	Previous ID	Origin
1	*Triticum urartu* Thum.	UR1	A^uA^u	Unknown	Unknown	Unknown
2	*Triticum urartu* Thum.	UR102	A^uA^u	Unknown	Unknown	Unknown
3	*Triticum urartu* Thum.	UR200	A^uA^u	Unknown	Unknown	Lebanon Talia
4	*Triticum urartu* Thum.	UR201	A^uA^u	Unknown	Unknown	Iraq
5	*Triticum urartu* Thum.	UR202	A^uA^u	Unknown	Unknown	Unknown
6	*Triticum urartu* Thum.	UR203	A^uA^u	Unknown	Unknown	Turkey
7	*Triticum urartu* Thum.	UR204	A^uA^u	Unknown	Unknown	Lebanon
8	*Triticum urartu* Thum.	UR205	A^uA^u	Unknown	Unknown	Unknown
9	*Triticum urartu* Thum.	UR206	A^uA^u	Unknown	Unknown	Lebanon
10	*Triticum urartu* Thum.	UR207	A^uA^u	Unknown	Unknown	Lebanon
11	*Triticum urartu* Thum.	UR208	A^uA^u	Unknown	Unknown	Unknown
12	*Triticum urartu* Thum.	UR209	A^uA^u	Unknown	Unknown	Izmir, Turkey
13	*Triticum boeoticum* Boiss.	BO1	A^bA^b	Unknown	Unknown	Unknown
14	*Triticum boeoticum* Boiss.	BO3	A^bA^b	P. 1. 355454	XM0943	East Germany
15	*Triticum boeoticum* Boiss.	BO5	A^bA^b	var. rutingrum	XM0944	America
16	*Triticum boeoticum* Boiss.	BO8	A^bA^b	Unknown	Unknown	Unknown
17	*Triticum boeoticum* Boiss.	BO9	A^bA^b	Unknown	Unknown	Unknown
18	*Triticum boeoticum* Boiss.	BO103	A^bA^b	Unknown	Unknown	Unknown
19	*Triticum boeoticum* Boiss.	BO104	A^bA^b	Unknown	Unknown	Unknown
20	*Triticum boeoticum* Boiss.	BO105	A^bA^b	Unknown	Unknown	Unknown
21	*Triticum monococcum* L.	MO2	A^mA^m	Unknown	Unknown	Unknown
22	*Triticum monococcum* L.	MO5	A^mA^m	var. bornemanni	XM0001	East Germany
23	*Triticum monococcum* L.	MDR001	A^mA^m	flavescens	Unknown	Algeria
24	*Triticum monococcum* L.	MDR025	A^mA^m	macedonicum; pseudoflavescens	Unknown	Crimea, Ukraine
25	*Triticum monococcum* L.	MDR026	A^mA^m	pseudomacedonicum	Unknown	Crimea, Ukraine
26	*Triticum monococcum* L.	MDR029	A^mA^m	flavescens	Unknown	Spain
27	*Triticum monococcum* L.	MDR035	A^mA^m	flavescens; vulgare	Unknown	Austria
28	*Triticum monococcum* L.	MDR036	A^mA^m	monococcum; pseudovulgare	Unknown	Czechoslovakia
29	*Triticum monococcum* L.	MDR037	A^mA^m	macedonicum	Unknown	Armenia
30	*Triticum monococcum* L.	ID27	A^mA^m	Unknown	Unknown	Iraq
31	*Triticum monococcum* L.	KU104-2	A^mA^m	Unknown	Unknown	Japan

32	*Triticum monococcum* L.	104006	A^mA^m	Unknown	Unknown	Unknown
33	*Triticum monococcum* L.	MO1	A^mA^m	Unknown	Unknown	Unknown
34	*Triticum monococcum* L.	MO4	A^mA^m	Unknown	Unknown	Unknown
35	*Triticum monococcum* L.	Pi39	A^mA^m	Unknown	Unknown	Unknown
36	*Aegilops speltoides*	Y597	SS	Unknown	Unknown	Unknown
37	*Aegilops speltoides*	Y590	SS	Unknown	Unknown	Unknown
38	*Aegilops speltoides*	Y2005	SS	Unknown	Unknown	Unknown
39	*Aegilops speltoides*	Y2012	SS	Unknown	Unknown	Unknown
40	*Aegilops speltoides*	Y2014	SS	Unknown	Unknown	Unknown
41	*Aegilops speltoides*	Y2018	SS	Unknown	Unknown	Unknown
42	*Aegilops speltoides*	Y2021	SS	Unknown	Unknown	Unknown
43	*Aegilops speltoides*	Y2042	SS	Unknown	Unknown	Unknown
44	*Aegilops speltoides*	Y2046	SS	Unknown	Unknown	Unknown
45	*Aegilops speltoides*	Y2049	SS	Unknown	Unknown	Unknown
46	*Aegilops speltoides*	Y2051	SS	Unknown	Unknown	Unknown
47	*Aegilops speltoides*	Y2056	SS	Unknown	Unknown	Unknown
48	*Aegilops longissima*	Y430	S^lS^l	Unknown	Unknown	Unknown
49	*Aegilops longissima*	Y431	S^lS^l	Unknown	Unknown	Unknown
50	*Aegilops longissima*	Y432	S^lS^l	Unknown	Unknown	Unknown
51	*Aegilops longissima*	Y433	S^lS^l	Unknown	Unknown	Unknown
52	*Aegilops longissima*	Y434	S^lS^l	Unknown	Unknown	Unknown
53	*Aegilops longissima*	Y435	S^lS^l	Unknown	Unknown	Unknown
54	*Aegilops sharonensis*	Ae29	$S^{sh}S^{sh}$	Unknown	RM0150	Unknown
55	*Aegilops sharonensis*	Y436	$S^{sh}S^{sh}$	Unknown	Unknown	Unknown
56	*Aegilops sharonensis*	Y437	$S^{sh}S^{sh}$	Unknown	Unknown	Unknown
57	*Aegilops sharonensis*	Y440	$S^{sh}S^{sh}$	Unknown	Unknown	Unknown
58	*Aegilops searsii*	Y446	S^sS^s	Unknown	Unknown	Unknown
59	*Aegilops searsii*	Y447	S^sS^s	Unknown	Unknown	Unknown
60	*Aegilops tauschii*	Y2262	DD	Unknown	Unknown	Unknown
61	*Aegilops tauschii*	Y2263	DD	Unknown	Unknown	Unknown
62	*Aegilops tauschii*	Y2265	DD	Unknown	Unknown	Unknown
63	*Aegilops tauschii*	Y2266	DD	Unknown	Unknown	Unknown
64	*Aegilops tauschii*	Y2269	DD	Unknown	Unknown	Unknown
65	*Aegilops tauschii*	Y2271	DD	Unknown	Unknown	Unknown

66	*Aegilops tauschii*	Y2272	DD	Unknown	Unknown	Unknown
67	*Aegilops tauschii*	Y2279	DD	Unknown	Unknown	Unknown
68	*Aegilops tauschii*	Y2281	DD	Unknown	Unknown	Unknown
69	*Aegilops tauschii*	Y2282	DD	Unknown	Unknown	Unknown
70	*Aegilops tauschii*	Y2286	DD	Unknown	Unknown	Unknown
71	*Aegilops tauschii*	Ae38	DD	Unknown	RM0160	France
72	*Aegilops tauschii*	Y2280	DD	Unknown	Unknown	Unknown
73	*Aegilops tauschii*	Ae35	DD	Unknown	RM0158	Canada
74	*Aegilops tauschii*	Y127	DD	Unknown	RM0188	East Germany
75	*Aegilops tauschii*	Y128	DD	Unknown	RM0189	East Germany
76	*Aegilops tauschii*	Y168	DD	Unknown	RM0190	America
77	*Aegilops tauschii*	Y169	DD	Unknown	RM0191	America
78	*Aegilops tauschii*	Y218	DD	Unknown	RM0231	Japan
79	*Aegilops tauschii*	Y219	DD	ssp. strangulata	RM0232	Japan
80	*Triticum dicoccoides* Koern.	DS1	AABB	Unknown	XM0945	France
81	*Triticum dicoccoides* Koern.	DS2	AABB	Unknown	Unknown	Unknown
82	*Triticum dicoccoides* Koern.	DS3	AABB	Unknown	Unknown	Unknown
83	*Triticum dicoccoides* Koern.	DS4	AABB	var. kotchanyum	XM0002	America
84	*Triticum dicoccoides* Koern.	DS5	AABB	Unknown	Unknown	Unknown
85	*Triticum dicoccoides* Koern.	DS8	AABB	var. spontanes Villosum	XM0005	East Germany
86	*Triticum dicoccoides* Koern.	DS9	AABB	var. kotschyi Abessinien	XM0003	West Germany
87	*Triticum dicoccoides* Koern.	DS10	AABB	1-38-55	XM0947	Canada
88	*Triticum dicoccoides* Koern.	DM1	AABB	Dhamar	XM0011	North Yemen
89	*Triticum dicoccum* L.	DM4	AABB	Amran	XM0008	North Yemen
90	*Triticum dicoccum* L.	DM6	AABB	Boll A	XM0009	North Yemen
91	*Triticum dicoccum* L.	DM12	AABB	Unknown	Unknown	Unknown
92	*Triticum dicoccum* L.	DM18	AABB	Var. farrum Alet., Abessinischer emmer	XM0017	East Germany
93	*Triticum dicoccum* L.	DM23	AABB	Unknown	Unknown	Unknown
94	*Triticum dicoccum* L.	DM36	AABB	Var. farrum Alet., N. p202	XM0021	East Germany
95	*Triticum dicoccum* L.	DM42	AABB	Weiber Amidonia	XM0029	East Germany
96	*Triticum dicoccum* L.	DM44	AABB	White Spring	XM0031	East Germany
97	*Triticum dicoccum* L.	DM46	AABB	Unknown	Unknown	Unknown
98	*Triticum dicoccum* L.	DM50	AABB	PD13	XM0949	Canada
99	*Triticum dicoccum* L.	DM51	AABB	REG1896	XM0950	Canada

100	*Triticum dicoccum* L.	DM135	AABB	Unknown	Unknown	Unknown
101	*Triticum dicoccum* L.	DM147	AABB	Unknown	Unknown	Unknown
102	*Triticum durum* Desf.	DR3	AABB	367210	XM0083	YugoslaviaYugoslavia
103	*Triticum durum* Desf.	DR4	AABB	5B×1D	XM0068	Italy
104	*Triticum durum* Desf.	DR13	AABB	6-1F r4-69	XM0069	Italy
105	*Triticum durum* Desf.	DR15	AABB	Damangchunmai	XM0032	Tibet, China
106	*Triticum durum* Desf.	DR53	AABB	Athena	XM0121	Italy
107	*Triticum durum* Desf.	DR146	AABB	Unknown	Unknown	Unknown
108	*Triticum durum* Desf.	DR148	AABB	Unknown	Unknown	Unknown
109	*Triticum durum* Desf.	DR333	AABB	Valgerado 1 - Geraldo 512	XM0683	Italy
110	*Triticum durum* Desf.	DR305	AABB	Unknown	Unknown	Unknown
111	*Triticum durum* Desf.	DR386	AABB	Unknown	Unknown	Unknown
112	*Triticum durum* Desf.	DR464	AABB	CMH79. 1159-DU-LAXJ	XM0198	Mexico
113	*Triticum durum* Desf.	DR487	AABB	DUR-10254F5	XM0249	Mexico
114	*Triticum durum* Desf.	DR484	AABB	Erpel"S"-Ruso	XM0262	Mexico
115	*Triticum durum* Desf.	DR492	AABB	Fg"S"-Dom"S"	XM0273	Mexico
116	*Triticum durum* Desf.	DR552	AABB	Rabi“S”-31810	XM0517	Mexico
117	*Triticum durum* Desf.	DR679	AABB	Unknown	Unknown	Unknown
118	*Triticum turgidum* L.	TG2	AABB	Sabtaibangbangnanmai	XM1101	Sichuan,China
119	*Triticum turgidum* L.	TG7	AABB	Youmangyumai	XM0775	Henan, China
120	*Triticum turgidum* L.	TG19	AABB	Fushoumai	XM0782	Shanxi, China
121	*Triticum turgidum* L.	TG23	AABB	Unknown	Unknown	Unknown
122	*Triticum turgidum* L.	TG27	AABB	Unknown	Unknown	Unknown
123	*Triticum turgidum* L.	TG29	AABB	Unknown	Unknown	Unknown
124	*Triticum turgidum* L.	TG33	AABB	Unknown	Unknown	Unknown
125	*Triticum turgidum* L.	TG39	AABB	Unknown	Unknown	Unknown
126	*Triticum carthlicum*	PS6	AABB	Var. fuliginosum Black Persian	XM1099	East Germany
127	*Triticum carthlicum*	PS8	AABB	Blauer Samtiger	XM0712	East Germany
128	*Triticum carthlicum*	PS9	AABB	Persischer	XM0713	East Germany
129	*Tiiticum polonicum* L.	PO1	AABB	Unknown	XM1100	Xinjiang, China
130	*Tiiticum polonicum* L.	PO6	AABB	Unknown	Unknown	Unknown
131	*Triticum turanicum* Jakubz.	TR1	AABB	Unknown	XM1097	Unknown
132	*Triticum turanicum* Jakubz.	TR5	AABB	Var. notabile	XM0708	East Germany
133	*Triticum araraticum* Jakubz.	AR1	AAGG	Unknown	XM0837	America

134	*Triticum araraticum* Jakubz.	AR3	AAGG	Unknown	XM0838	America
135	Landrace	Baihuamai	AABBDD	Unknown	ZM008598	Xingyi, Guizhou
136	Landrace	Baimaizi	AABBDD	Unknown	ZM008547	Deyang, Sichuan
137	Landrace	Jiangxizao	AABBDD	Unknown	ZM003464	Shangcheng, Henan
138	Landrace	Baimangmai	AABBDD	Unknown	ZM008732	Jinping, Guizhou
139	Landrace	Sanyuehuang	AABBDD	Unknown	ZM002685	Wenxian, Henan
140	Landrace	Qiangchangmai	AABBDD	Unknown	ZM003793	Changan, Shaanxi
141	Landrace	Lanhuamai	AABBDD	Unknown	ZM005017	Jingchuan, Gansu
142	Landrace	Hongdongmai	AABBDD	Unknown	ZM005188	Tacheng, Xinjiang
143	Landrace	Mahuaban	AABBDD	Unknown	ZM004422	Jilin
144	Landrace	Hongjinmai	AABBDD	Unknown	ZM020735	Pingliang, Gansu
145	Landrace	Baidongmai	AABBDD	Unknown	ZM005439	Qitai, Xinjiang
146	Landrace	Chinese Spring	AABBDD	Unknown	ZM005452	Sichuan
147	Landrace	Baimazha	AABBDD	Unknown	ZM020808	Unknown
148	Landrace	Youmangbaifu	AABBDD	Unknown	ZM004418	Unknown
149	Landrace	Xiaofoshou	AABBDD	Unknown	ZM002686	Unknown
150	Landrace	Youmangsaogudan	AABBDD	Unknown	ZM002659	Unknown
151	Modern cultivar	Zhengmai9023	AABBDD	Unknown	Unknown	Henan
152	Modern cultivar	Jinmai8	AABBDD	Unknown	ZM009368	Shanxi
153	Modern cultivar	Yannong15	AABBDD	Unknown	ZM015719	Shandong
154	Modern cultivar	Lvhan328	AABBDD	Unknown	ZM014050	Shanxi
155	Modern cultivar	Lankao906	AABBDD	Unknown	ZM025358	Henan
156	Modern cultivar	Xuzhou22	AABBDD	Unknown	ZM022308	Jiangsu
157	Modern cultivar	Nongda139	AABBDD	Unknown	ZM009018	Beijing
158	Modern cultivar	Jinyang60	AABBDD	Unknown	ZM009648	Shaanxi
159	Modern cultivar	Jinmai11	AABBDD	Unknown	ZM014430	Shanxi
160	Modern cultivar	Jimai19	AABBDD	Unknown	ZM013873	Hebei
161	Modern cultivar	Lumai9	AABBDD	Unknown	ZM015838	Shandong
162	Modern cultivar	Beijing15	AABBDD	Unknown	ZM008970	Beijing
163	Modern cultivar	Mingxian169	AABBDD	Unknown	ZM009379	Shanxi
164	Modern cultivar	Pan86001-3	AABBDD	Unknown	ZM024490	Guizhou

Table S3

Species	Accession ID/name	KW (mm)	TKW (g)
T. urartu	UR1	0.76	9.88
T. urartu	UR203	0.81	10.17
T. urartu	UR206	0.91	10.34
T. urartu	UR209	0.96	9.93
T. boeoticum	BO1	1.47	14.25
T. boeoticum	BO3	1.52	12.97
T. boeoticum	BO5	1.41	13.56
T. boeoticum	BO9	1.40	14.63
T. monococcum	MDR035	1.39	20.52
T. monococcum	MDR036	1.34	19.87
T. monococcum	MDR037	1.78	19.98
T. monococcum	ID27	1.82	20.11
T. monococcum	KU104-2	1.66	21.34
T. monococcum	104006	1.96	18.96
T. monococcum	PI39	1.69	20.67
T. monococcum	MDR001	2.12	20.51
T. monococcum	MDR025	1.29	18.76
T. monococcum	MDR026	1.26	18.88
T. monococcum	MDR029	1.49	19.25
T. monococcum	MO1	1.85	20.22
T. monococcum	MO4	2.09	20.21
T. monococcum	MO101	1.60	18.14
Ae. speltoides	Y605	1.74	4.56
Ae. speltoides	Y2002	1.78	4.47
Ae. speltoides	Y2003	1.73	5.13
Ae. speltoides	Y2022	1.77	5.24
Ae. speltoides	Y2030	1.59	4.36
Ae. speltoides	Y2008	1.66	4.18
Ae. speltoides	Y2017	1.70	5.01
Ae. speltoides	Y2019	1.84	5.17
Ae. longissima	Y430	1.79	5.23
Ae. longissima	Y432	1.77	5.65
Ae. longissima	Y433	1.47	4.89
Ae. longissima	Y434	2.04	5.78
Ae. longissima	Y2147	1.62	4.97
Ae. longissima	Y2144	1.88	5.06
Ae. tauschii	Y2266	2.69	13.03
Ae. tauschii	Y2269	2.95	13.28
Ae. tauschii	Y2271	3.11	14.56
Ae. tauschii	Y2281	2.89	13.26
Ae. tauschii	Y2280	3.39	14.02
T. dicoccoides	DS1	2.34	39.43
T. dicoccoides	DS4	2.46	28.80
T. dicoccoides	DS5	2.45	16.90
T. dicoccoides	DS8	2.24	31.10
T. dicoccum.	DM12	2.36	35.31

T. dicoccum.	DM23	2.63	33.43
T. dicoccum.	DM46	2.13	27.62
T. dicoccum.	DM147	2.89	28.45
T. durum	DR3	3.11	29.73
T. durum	DR53	2.80	35.35
T. durum	DR146	2.85	42.24
T. durum	DR148	2.81	37.91
T. durum	DR305	2.76	29.82
Landrace	Baihuamai	2.75	21.54
Landrace	Baimaizi	3.22	29.26
Landrace	Jiangxizao	2.98	28.80
Landrace	Baimangmai	2.93	27.38
Landrace	Sanyuehuang	2.98	25.69
Landrace	Qiangchangmai	2.95	25.23
Landrace	Lanhuamai	2.99	25.56
Landrace	Hongdongmai	2.88	29.68
Landrace	Mahuaban	2.68	23.06
Landrace	Hongjinmai	2.73	23.81
Landrace	Baidongmai	2.83	24.87
Landrace	Chinese Spring	3.03	27.32
Landrace	Baimazha	2.67	27.50
Landrace	Youmangbaifu	3.30	37.74
Landrace	Xiaofoshou	3.15	35.48
Landrace	Youmangsaogudan	3.19	38.01
Modern cultivar	Zhengmai9023	3.12	43.15
Modern cultivar	Jinmai8	3.33	43.55
Modern cultivar	Yannong15	3.35	36.37
Modern cultivar	Lvhan328	3.29	37.06
Modern cultivar	Lankao906	3.44	49.77
Modern cultivar	Xuzhou22	3.68	53.82
Modern cultivar	Nongda139	2.93	35.53
Modern cultivar	Jinyang60	3.15	25.86
Modern cultivar	Jinmai11	3.25	43.50
Modern cultivar	Jimai19	3.15	34.04
Modern cultivar	Lumai9	3.57	47.34
Modern cultivar	Beijing15	3.03	32.40
Modern cultivar	Mingxian169	3.03	30.18
Modern cultivar	Pan86001-3	3.71	51.89

Table S4

Primer set	Primer sequence	Amplified target	Tm (°C)
TaGW2-P-1	Forward: CGTTACCTCTGGTTTGGGTGTCGTG	Promoter amplification	60
	Reverse: GCGGCACTCTACGGCAGAACAAAT		
TaGW2-P-2	Forward: AAGGGGAGATACATGAGCTAAGC	Promoter amplification	60
	Reverse: AGGAGTACAACGTCCGCTAAGGG		
TaGW2-1	Forward: ATGGGGAACAGAATAGGAGGGAG	Coding amplification	56
	Reverse: GACATTCAGGGTCAGAAGTCCAG		
TaGW2-2	Forward: TGACAGCTACTCTAAAAGCAGGGAG	Coding amplification	55
	Reverse: ATCTCCTTACTTGCCACAGCCAC		
TaGW2-3	Forward: TGATGGTTAGGCAGGGTTAGAAG	Coding amplification	55
	Reverse: GCTTCCATAACCATCATGTCCTC		
TaGW2-4	Forward: CGAGCCAGAATAAAGTTGAGGTTC	Coding amplification	57
	Reverse: TGACAGCTACTCTAAAAGCAGGGAG		
TaGW2-6A-RT	Forward: CTGCGGAAAGTTCACCAGATAG	*TaGW2-6A* Real-time PCR	63
	Reverse: TGTCAGCAAAAGGCAACGGTA		
TaGW2-6B-RT	Forward: GATAGCTGGAGCGGGATAGCAT	*TaGW2-6B* Real-time PCR	63
	Reverse: TCAGTAACAGGCAACGGTGGAG		
TaGW2-6D-RT	Forward: GTATAGGAAATCCTGCTTGTGGG	*TaGW2-6D* Real-time PCR	63
	Reverse: TGTAAGAGAAATCCATGCTTGC		
Actin	Forward: CGATTCAGAGCAGCGTATTGTTG	Actin	63
	Reverse: AGTTGGTCGGGTCTCTTCTAAATG		

Table S5

A

	DI	TE	LA
DI			
TE	0.000		
LA	0.000	0.005	
MC	0.000	0.009	0.035

B

	DI	TE	LA
DI			
TE	0.000		
LA	0.000	0.000	
MC	0.000	0.000	0.015

C

	DI	LA
DI		
LA	0.000	
MC	0.000	0.029

D

	DI	TE	LA
DI			
TE	0.000		
LA	0.000	0.000	
MC	0.000	0.000	0.048

E

	DI	TE	LA
DI			
TE	0.000		
LA	0.000	0.026	
MC	0.000	0.019	0.042

F

	DI	LA
DI		
LA	0.000	
MC	0.000	0.029

DI: diploid; TE: tetraploid; LA: landrace; MC: modern cultivar

Table S6

Gene	Region	Diploids		Tetraploids		Hexaploids	
		θ(10^{-3})	Tajima's *D*	θ(10^{-3})	Tajima's *D*	θ(10^{-3})	Tajima's *D*
	Promoter	4.19	2.67828**	1.93	-1.03171	0.96	2.42128**
TaGW2-6A	Gene	2.20	2.03875*	0.61	1.3324	0.04	0.67135
	Total region	2.61	2.35422*	0.93	1.4765	0.20	3.25404**
	Promoter	17.98	2.49508**	2.55	-1.11849	1.04	1.91408
TaGW2-6B	Gene	5.66	2.02828*	1.03	1.5864	0.19	1.71112
	Total region	7.39	2.79353**	1.29	1.6878	0.33	1.85298
	Promoter	3.39	0.86383	—	—	—	—
TaGW2-6D	Gene	1.50	0.86383	—	—	—	—
	Total region	1.90	1.54697	—	—	—	—

θ, Watterson estimator; Tajima's *D*, test for neutral selection.
*Significant at $P<0.05$; **significant at $P<0.01$.

Table S7

Gene	Ploidy	Species	Relative transcription level (mean ±S.E)	Average	Kernel width (mm) (mean ±S.E)	Average	1000 kernel weight (g) (mean ±S.E)	Average
TaGW2-6A	Diploid	*T. urartu*	2.899±0.431	3.128±0.598a	0.858±0.100	1.552±0.086a(A)	10.176±0.107	14.560±1.738a
		T. boeoticum	3.772±1.822		1.432±0.035		13.780±0.618	
		T. monococcum L.	2.652±0.407		1.667±0.076		19.723±3.551	
	Tetraploid	*T. dicoccoides*	1.557±0.685	1.281±0.235a	2.350±0.110	2.603±0.109ab(AB)	29.200±4.618	31.824±1.507a
		T. dicoccum	1.305±0.317		2.502±0.164		28.876±2.102	
		T. durum	1.035±0.088		2.912±0.104		34.936±2.003	
	Hexaploid	Landraces	1.559±0.732	1.148±0.278a	2.950±0.002	3.155±0.025b(B)	33.060±0.423	35.846±0.434a
		Modern cultivars	0.902±0.120		3.200±0.003		40.810±0.334	
TaGW2-6B	Diploid	*Ae. speltoides*	7.055±0.674	5.188±0.913a(A)	1.724±0.031	1.741±0.037a(A)	4.578±0.613	5.085±0.356a(A)
		Ae. longissima	2.316±1.226		1.757±0.068		5.592±0.043	
		Ae. sharonensis	2.598±0.513		1.690±0.056		5.469±0.216	
	Tetraploid	*T. dicoccoides*	2.609±0.355	2.426±0.193b(B)	2.350±0.110	2.603±0.109b(B)	29.200±4.618	31.824±1.507b(B)
		T. dicoccum	2.314±0.451		2.503±0.164		28.876±2.102	
		T. durum	2.378±0.295		2.912±0.104		34.936±2.003	
	Hexaploid	Landraces	1.611±0.131	1.434±0.139c(B)	2.950±0.002	3.155±0.025b(AB)	33.060±0.423	35.846±0.434b(B)
		Modern cultivars	1.138±0.168		3.200±0.003		40.810±0.334	
TaGW2-6D	Diploid	*Ae.tauschii*	5.734±1.167	5.734±1.167a	3.140±0.250	3.140±0.250a	13.845±0.382	13.845±0.382a
	Hexaploid	Landraces	1.438±0.219	1.372±0.078b	2.950±0.002	3.155±0.025a	33.060±0.423	35.846±0.434b
		Modern cultivars	1.333±0.038		3.200±0.003		40.810±0.334	

Capital and small letters show the significance level at $P < 0.01$ and $P < 0.05$ compared between ploidy levels for each trait, respectively.

Table S8

Ploidy	Gene	Overall transcription levels (Mean±S.E)	Mean kernel width (mm) (Mean±S.E)	Mean 1000-kernel weight (g) (Mean±S.E)
Diploid	*TaGW2-6A*	3.128±0.598a	1.552±0.086a	14.560±1.738ab
	TaGW2-6B	5.188±0.913b	1.741±0.037a(A)	5.085±0.356a(A)
	TaGW2-6D	5.734±1.167b	3.140±0.250ab	13.845±0.382ab
Tetraploid	*TaGW2-6A*	3.426±1.225a	2.603±0.109ab	31.824±1.507b
	TaGW2-6B			
Hexaploid	*TaGW2-6A*	3.530±0.876a	3.155±0.025b(B)	35.846±0.434b(B)
	TaGW2-6B			
	TaGW2-6D			

Capital and small letters show the significance level at $P<0.01$ and $P<0.05$ compared between ploidy levels for each trait, respectively.
